# Inference of Low and High-Grade Glioma Gene Regulatory Networks Delineates the Role of Rnd3 in Establishing Multiple Hallmarks of Cancer

**DOI:** 10.1371/journal.pgen.1005325

**Published:** 2015-07-01

**Authors:** Kim Clarke, Thomas Daubon, Nil Turan, Fabienne Soulet, Maihafizah Mohd Zahari, Katie R. Ryan, Sarah Durant, Shan He, John Herbert, John Ankers, John K. Heath, Rolf Bjerkvig, Roy Bicknell, Neil A. Hotchin, Andreas Bikfalvi, Francesco Falciani

**Affiliations:** 1 Centre for Computational Biology and Modelling (CCBM), Institute of Integrative Biology, University of Liverpool, Liverpool, United Kingdom; 2 INSERM U1029, University Bordeaux, Pessac, France; 3 NorLux Neuro-Oncology, Department of Biomedicine, University of Bergen, Bergen, Norway; 4 Medical School, University of Birmingham, Birmingham, United Kingdom; 5 School of Biosciences, University of Birmingham, Birmingham, United Kingdom; 6 Computer Science, University of Birmingham, Birmingham, United Kingdom; University of Texas MD Anderson Cancer Center, UNITED STATES

## Abstract

Gliomas are a highly heterogeneous group of brain tumours that are refractory to treatment, highly invasive and pro-angiogenic. Glioblastoma patients have an average survival time of less than 15 months. Understanding the molecular basis of different grades of glioma, from well differentiated, low-grade tumours to high-grade tumours, is a key step in defining new therapeutic targets. Here we use a data-driven approach to learn the structure of gene regulatory networks from observational data and use the resulting models to formulate hypothesis on the molecular determinants of glioma stage. Remarkably, integration of available knowledge with functional genomics datasets representing clinical and pre-clinical studies reveals important properties within the regulatory circuits controlling low and high-grade glioma. Our analyses first show that low and high-grade gliomas are characterised by a switch in activity of two subsets of Rho GTPases. The first one is involved in maintaining normal glial cell function, while the second is linked to the establishment of multiple hallmarks of cancer. Next, the development and application of a novel data integration methodology reveals novel functions of RND3 in controlling glioma cell migration, invasion, proliferation, angiogenesis and clinical outcome.

## Author Summary

Gliomas are aggressive brain tumours that are invasive, heterogeneous, refractory to treatment and show poor survival rates. Surgical resection and chemotherapy can increase patient survival but ultimately the disease is fatal. Multiple grades of glioma exist, with lower grades associated to better prognosis. While the majority of high-grade gliomas occur *de novo*, it is common that low-grade gliomas progress to the more aggressive form known as glioblastoma. In this article, we have shown that by combining advanced network biology approaches with the right experimental models, we are able to reveal novel regulatory circuits controlling multiple hallmarks of glioma. Through analysis of multiple network models representing protein-protein interaction or gene co-expression data we have revealed a switch in the role of regulatory Rho GTPases between low and high-grade gliomas. Amongst these, we show that RND3 is up-regulated in glioblastomas and is a key regulator of tumour proliferation, migration and invasion. We confirm that expression and genomic copy number of RND3 are predictive of clinical outcome, suggesting that changes in the activity of this particular Rho GTPase could be an early event associated to transformation and tumour expansion.

## Introduction

Gliomas are brain tumours originating from the glial cells and neural stem cells that surround and support neurons [1]. They are classified on the basis of their clinical and histopathological characteristics in four grades with progressively more severe features. Grade I and II gliomas (astrocytomas, oligodendrogliomas and oligoastrocytomas) are considered relatively benign, well-differentiated tumours and have 5 year survival rates of 59.9% [2]. Amongst patients diagnosed with low-grade gliomas, approximately 70% progress to grade IV glioblastoma multiforme (GBM) within 5–10 years of diagnosis [3]. *De novo* GBM constitute the majority of grade IV glioma and are powerful inducers of angiogenesis, highly proliferative and invasive. They are largely resistant to treatment and have poor prognosis with two years survival rates as low as 3.3% [4].

A number of studies have identified key genomic alterations in GBM able to induce transformation in non-tumorigenic cells such as mutations within EGFR [5] [6] and PDGFRA [7]. A molecular classification for GBM has been proposed subdividing the tumours based on their molecular profile into 4 groups. This includes the classical type (EGFR amplification, CDKN2A deletion), proneural type (PDGFRA amplification, PTEN deletion), the mesenchymal (NF1 deletion) and the neural type [8]. However, angiogenic and invasive phenotypes are observed across the different groups, making this classification unsatisfactory. For example, EGFR amplification reminiscent of the classical type has been shown to drive invasive growth [9]. Amplification and overexpression of EGFR leads to activation of Ras GTPase and Akt signalling pathways controlling cell growth, differentiation and survival of tumour cells [10] [11].

The complexity of the factors involved in the biology of gliomas makes it difficult to develop a comprehensive model underlying GBM progression. Here we address this important challenge by integrating functional genomics datasets representing existing knowledge, clinical studies and *in vivo* and *in vitro* glioma models.

We first show that network modules derived from a comprehensive integration of protein interaction databases and defined by a high density of genes differentially expressed between low and high-grade gliomas are consistent with the hypothesis that Rho GTPases may be part of a key regulatory mechanism controlling hallmarks of high-grade glioma. A key feature of GBM is invasion of tumour cells into the surrounding brain tissue and members of the Rho GTPase family known to control actin cytoskeleton dynamics and cell migration have been implicated in the survival and invasion of tumour cells [12] [13], [14]. In addition, RhoA expression correlates to tumour grade in astrocytomas [15]. We reconstruct stage specific gene co-expression networks and analyse the connectivity profile of Rho GTPases. This reveals that regulatory Rho GTPases separates in two groups, one active in low and the other in high-grade gliomas. The functional profile of the putative targets of these two sub-sets predicts the functional differences observed between low and high-grade glioma.

Further characterisation of a high-grade glioma transcriptional network highlights a pivotal role of the Rho GTPase RND3 (also known as RhoE, Entrez: 390) in controlling tumour proliferation, migration and invasion. Ultimately, the clinical relevance of this regulatory network is proving that copy number variation in the RND3 gene is predictive of clinical outcome.

## Results

### Overview of the analysis and validation strategy

**Fig 1:**
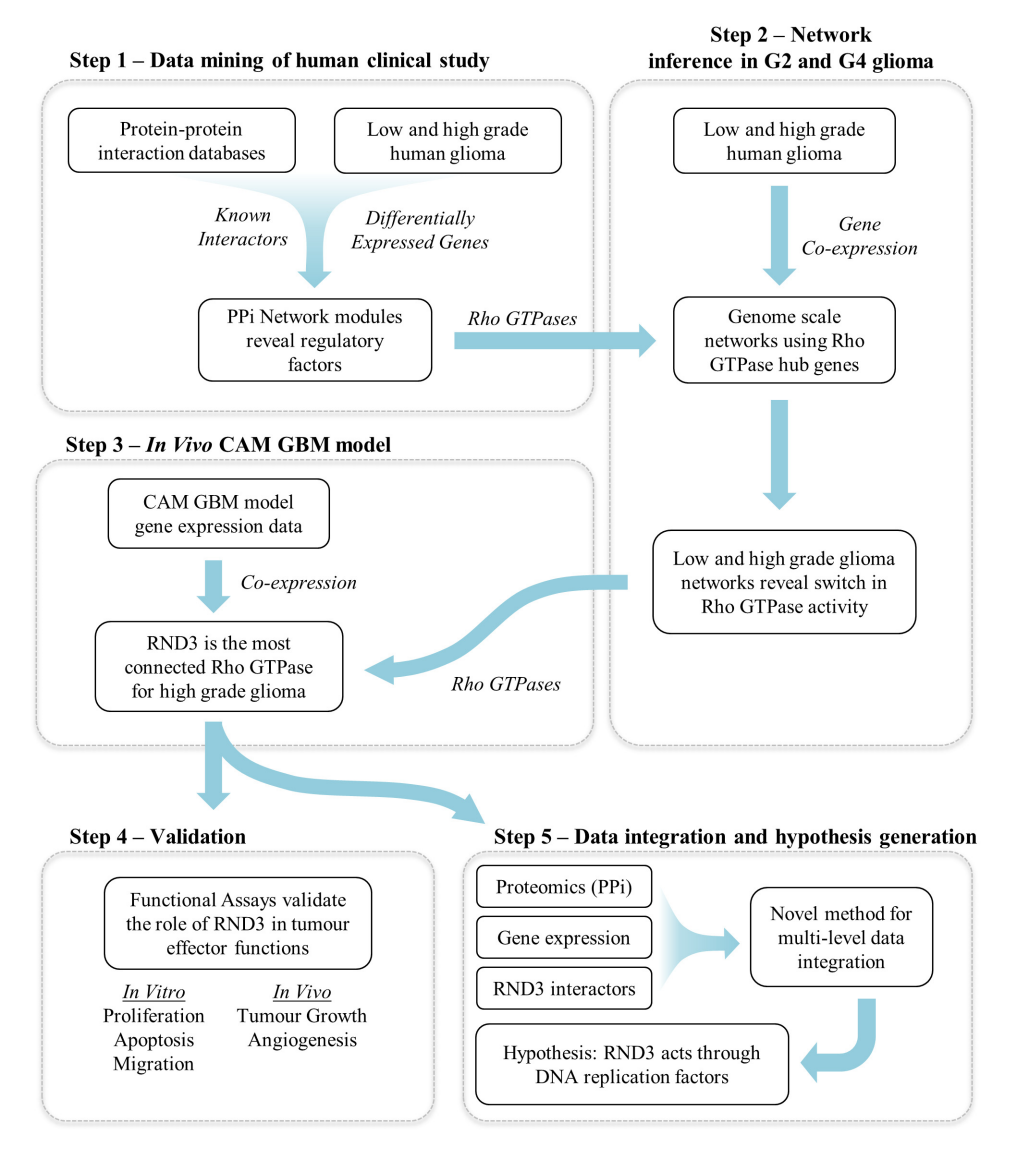
Overview of the analysis and validation strategy.

Our study is based on a complex data analysis workflow which includes several complementary reverse engineering techniques to address the important challenge of generating and validating hypotheses outlining the main factors underlying the control and maintenance of glioma stage. The strategy we followed, which is summarised in Fig 1, is based on several cycles of data acquisition, computational analysis, hypothesis generation and experimental validation.

The workflow consisted of five distinct but interconnected steps.

Step 1- Integration of protein-protein interaction networks with gene expression data derived from a human clinical study: This represents low and high-grade gliomas and were used to modularise a large network of known human protein-protein interactions (PPi). The analysis of these modules identified a unique sub-network of regulatory factors, which represented a number of Rho GTPases.

Step 2- Revealing the linkage between subsets of Rho GTPases and glioma grade: Genome scale gene expression data were used to construct co-expression networks centred on the regulatory Rho GTPases identified in step 1. This revealed a switch in activity of Rho GTPases between grade II and grade IV glioma.

Step 3- Reverse engineering of regulatory networks from an *in vivo* model:

We used the experimental glioma model in the chick chorioallantoic membrane [16] which revealed regulation of Rho GTPases in the angiogenic and invasive phase of glioma development. Network analysis revealed RND3 as the most connected factor for high-grade glioma.

Step 4- Validation step: The predictions from the network analysis were validated through functional assays (apoptosis, proliferation, migration and angiogenesis *in vitro* and *in vivo*).

Step 5- Development of data integration strategy: We developed a novel, multi-level data integration pipeline in order to shed light on the possible mechanisms of RND3 control of tumour function. This revealed a link between RND3 and DNA replication factors such as MCM3, which we validate *in vitro*.

### Modularization of the human proteome identifies a cluster of GTPases with potential regulatory activity in high-grade glioma

In order to gain insight in the mechanisms underlying the pathophysiology of human glioma, we first developed an interaction network representing genes differentially expressed between grade II astrocytoma and grade IV glioblastoma. We then applied a modularization procedure to identify sub-networks of highly interconnected proteins, thus capturing important biological networks potentially representative of the differences between low and high-grade glioma.

**Fig 2:**
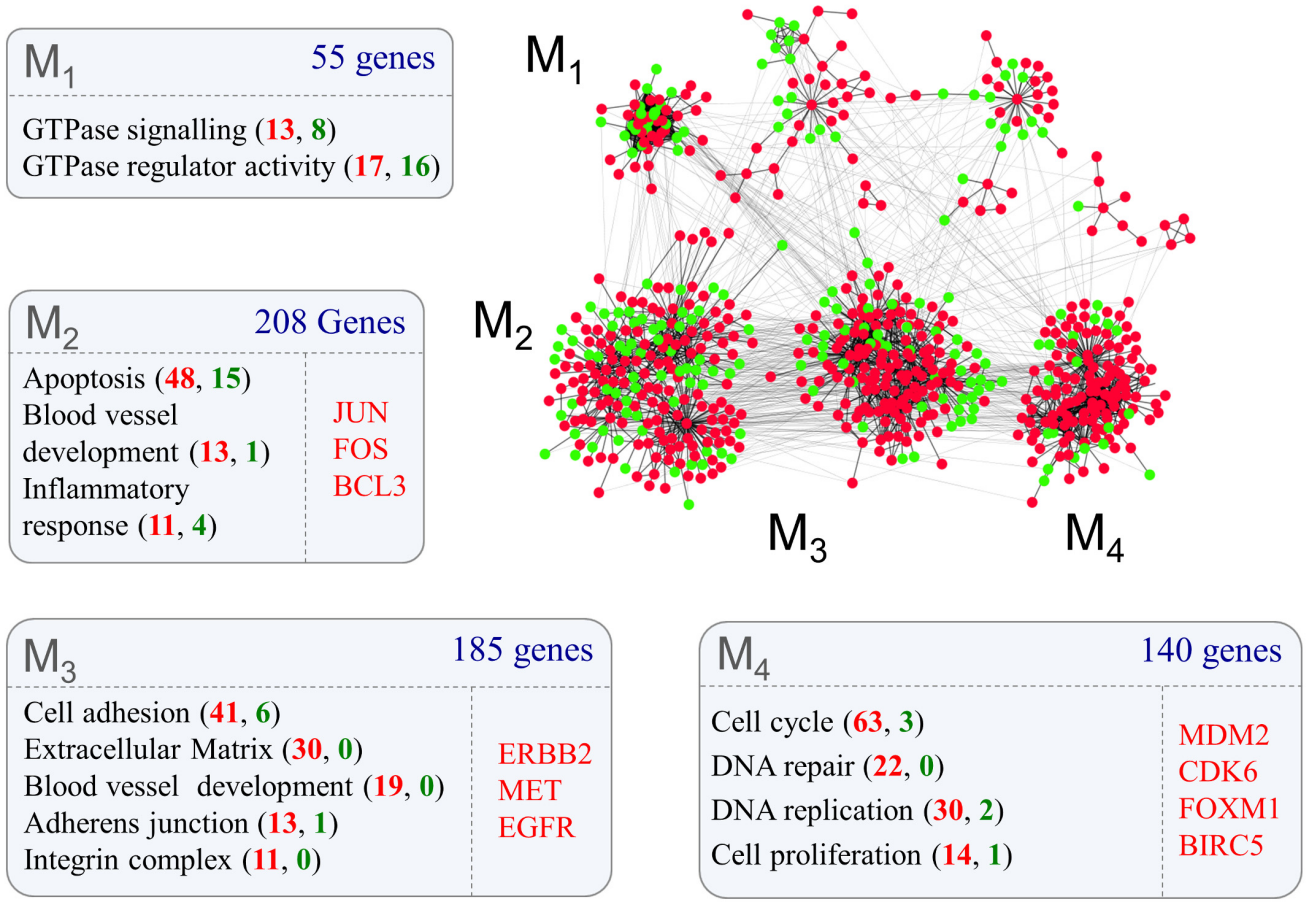
Protein-protein interaction networks representing glioma grade transition reveal functionally distinct modules. Proteins for which mRNA abundance is higher (red nodes) or lower (green nodes) in grade IV glioma form highly interconnected modules (network modules M_1_-M_4_) defined by known protein-protein interactions. Gene ontology analysis of each module (box M_1_-M_4_) reveal functional terms enriched within clusters (false discovery rate < 10%). The number of genes mapped to each Gene Ontology term is shown in brackets and are coloured red if they are up or green if they are down-regulated in grade IV glioma. Individually listed genes are known cancer oncogenes within each cluster, of which all were up-regulated in grade IV glioma.

**Fig 3:**
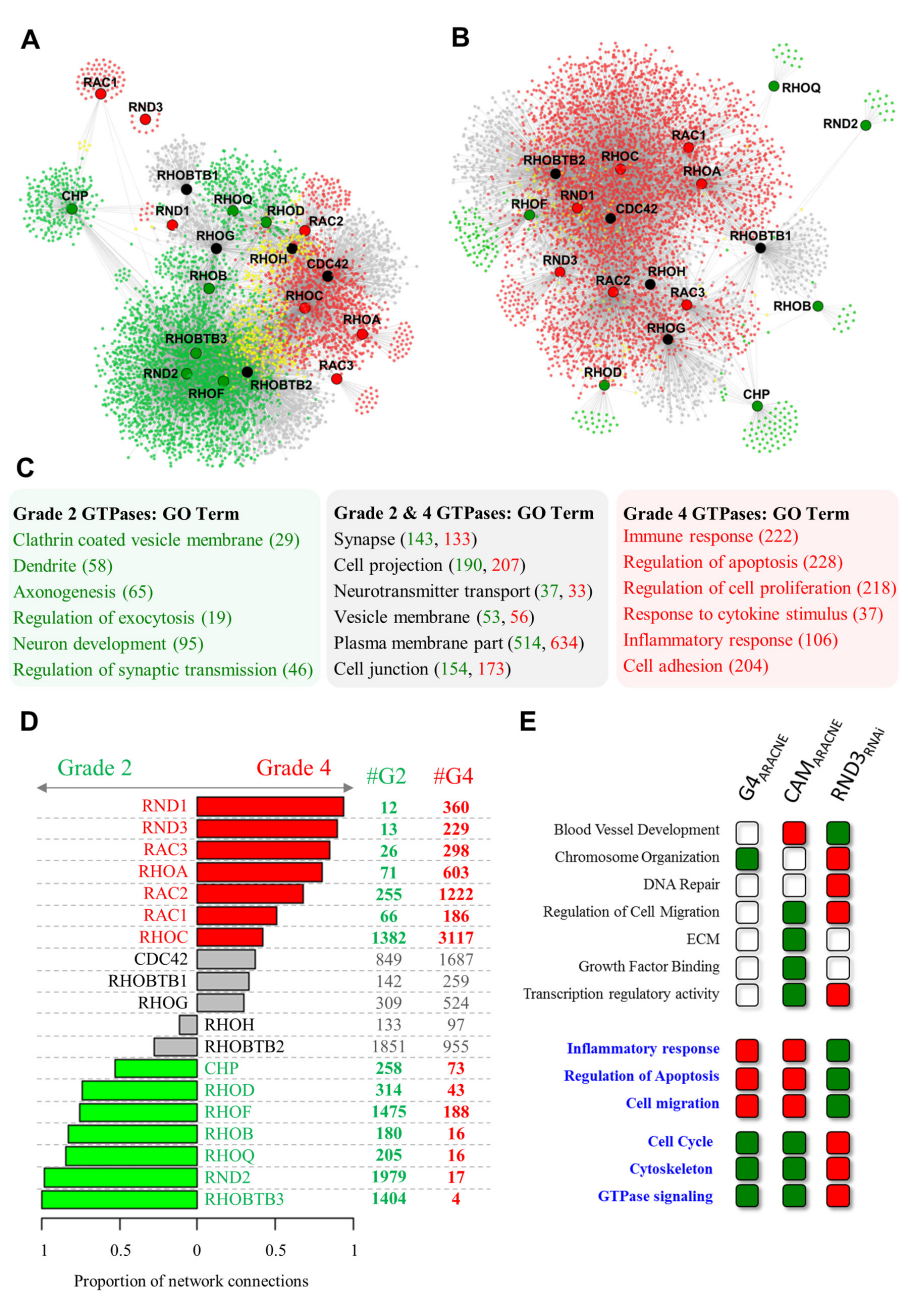
Transcriptional networks reveal two groups of Rho GTPases with divergent connectivity in grade II and grade IV glioma. **A, B.** Transcriptional co-expression networks representing the neighbourhood of Rho GTPases in (**A**) grade II (5973 genes) and (**B**) grade IV (5621 genes) glioma. **C.** Gene Ontology terms enriched within genes connected to grade II specific Rho GTPases or grade IV specific Rho GTPases (determined by connectivity, shown in panel D). False discovery rate < 10%, number of genes indicated in brackets (grade II: green, grade IV: red). **D.** Two distinct subsets of Rho GTPases show a significantly higher number of connections in either grade II (green) or grade IV (red) glioma. Differences in connectivity were expressed as the proportion of grade II to grade IV connections. Rho GTPases with an absolute difference between tumour grades > 0.4 were considered grade specific. **E.** Transcriptional profiling of U87 siRND3 cells induces a functional profile consistent with the network analysis in primary and CAM implanted tumours. Each Gene Ontology term represents genes within that category which are linked to RND3 (connected to RND3 in the grade IV glioma network and/or connected to RND3 in the CAM ARACNE network and/or differentially expressed in U87 siRND3 cells). These genes are either positively (red boxes) or negatively (green boxes) correlated with expression of RND3 or up-regulated (red boxes) or down-regulated (green boxes) in U87 siRND3 cells. Gene Ontology terms matched to all 3 experiments are highlighted in blue. Genes in networks (**A**) and (**B**) are coloured according to whether they are connected to Rho GTPases with grade specific connectivity in grade IV glioma (red nodes), grade II glioma (green nodes), or both grade II and grade IV (yellow nodes). Rho GTPases in networks (**A**) and (**B**) are coloured according to the grade specific connectivity shown in panel D (red: grade IV, green: grade II, black: non-specific).

The procedure identified four main modules, with a highly statistically significant functional enrichment profile (Fig 2). The entire list of significantly enriched Gene Ontology terms can be found in S1 Dataset. Interestingly, the three largest modules (**M**_**2-4**_) mainly comprised genes up regulated in grade IV gliomas and were enriched in typical effector functions in cancer. More precisely, the largest of the effector modules (**M**_**2**_) was enriched in genes linked to *apoptosis*, *blood vessel development* and *inflammatory response* and included the oncogenes JUN, FOS and BCL3 (for reviews of known oncogenes see [17], [18]). The second largest module (**M**_**3**_) included genes involved in *cell adhesion*, *extracellular matrix*, *blood vessel development*, *adherens junctions* and *integrin complex* and also included the oncogenes ERBB2, MET and EGF1. Effector module **M**_**4**_ was predominantly enriched with proliferation related functions such as *cell cycle*, *DNA repair* and *DNA replication* and the oncogenes MDM2, CDK6, FOXM1 and BIRC5. We noticed that the smallest module (**M**_**1**_) comprised mainly proteins linked to GTPase signalling (41/54), suggesting that this class of proteins may represent more important regulators of tumour effector functions than previously anticipated. By examining the functional enrichment profile of this cluster we discovered that the only enriched family of GTPases was the Rho family with 5 members (CDC42, RHOJ, RAC2, RHOC, and RND3) (False Discovery Rate < 1.93^−6^). This observation is consistent with the pro-tumour function of CDC42, RHOG, RAC1 and RHOA in glioma [14] [13] [19] [20] [15] [21] [22]. However, our model suggests a broader role of GTPases in glioma than previously thought. We therefore tested this hypothesis by inferring the structure of grade II (Fig 3A) and grade IV (Fig 3B) glioma transcriptional networks in the neighbourhood of all Rho GTPases.

### Reverse engineering grade II and grade IV glioma transcriptional networks identifies two functionally distinct subsets of GTPases of the Rho family

We discovered that genes encoding for Rho GTPases were separated in two groups when characterized by tumour grade-specific connectivity profiles (Fig 3D). Seven Rho GTPases (RND1, RND3, RAC3, RHOA, RAC2, RAC1 and RHOC) showed a significantly greater connectivity in grade IV glioma and seven (CHP, RHOD, RHOF, RHOB, RHOQ, RND2, RHOBTB3) showed greater connectivity in grade II glioma. In order to validate this differential connectivity we used expression data from grade II and grade IV glioma within the Cancer Genome Atlas database. 16 Rho GTPases could be matched between the original data and TCGA grade II and grade IV datasets, of which 13 (81%) showed the same trend as the original analysis (S2 Dataset).

Remarkably, Rho GTPases with a higher number of connections in grade IV tumours were correlated to genes in the grade IV networks with a functional profile that included many of the effector functions associated with high-grade glioma (*immune response*, *regulation of apoptosis*, *regulation of cell proliferation*, *response to cytokine stimulus*, *inflammatory response*, *cell adhesion)* (Fig 3C). On the other hand, in the grade II networks targets of Rho GTPases with a higher proportion of connections in grade II glioma showed a functional profile consistent with glial cells (putative target genes for this group of regulators were enriched with *clathrin coated vesicle membrane*, *dendrite*, *axonegensis*, *regulation of exocytosis*, *neuron development and regulation of synaptic transmission* functional terms) (Fig 3C). The full list of Gene Ontology terms from the analysis of the grade II and grade IV networks can be found in S3 Dataset. The functional profiles of the targets of the two groups of Rho GTPases also contained a subset of similar functional terms such as *synapse*, *cell projection*, *neurotransmitter transport*, *vesicle membrane*, *cell junction*. This suggests that in grade IV gliomas the link between Rho GTPases and normal glial function is only partially interrupted.

Interestingly, we found that although most (15/19) of the Rho GTPases were differentially expressed between grade II and grade IV gliomas, there was not a clear trend in the direction of change (S2 Fig). Additionally, none of the Rho GTPases were differentially expressed between grade II and grade III gliomas (S2 Fig). Overall, this supports the hypothesis that distinct subsets of Rho GTPases are potentially important players in grade IV gliomas.

### An *in vivo* model of glioma development validates the predictions of the inferred networks and identifies RND3 as a key regulatory molecule

In order to validate the networks developed from the clinical study, we implanted a grade IV glioma derived cell line (U87MG) in the chicken egg chorioallantoic membrane (CAM) and followed the transcriptional profile of tumour cells for the first 5 days post implantation with 10 equally spaced time points. In this well established *in vivo* model, tumours implanted on the CAM form avascular, solid tumours which stimulate angiogenesis and become vascularised within 48 hours [16].

We found 2,999 unique genes differentially expressed during 5 days of tumour growth. We used these genes to build a high level map representing the dynamics of transcriptional changes in the developing tumour. A gene clustering procedure identified 14 distinct clusters which were stratified according to their expression profiles (S3 Fig) (for gene lists of each cluster and the Gene Ontology analysis see S4 Dataset). Functional profiling of the early transcriptional response post-implantation (up to 12hrs) revealed an increase in expression of *extracellular matrix components*. The intermediate transcriptional response (13 to 24hrs) was characterised by a wave of transcriptional repression related to the *inflammatory response*, *regulation of apoptosis*, *lipid biosynthetic process* and *lysosomes* functions. These included the extracellular matrix remodelling genes, BMP2 and BMP6, chemokine signalling genes CCR1 and CCL20 and the inflammatory cytokine IL1A (S3 Fig). Consistent with the development of a fully vascularized tumour at 48 hours post-implantation we saw a dramatic increase in the expression of genes related to the *cell cycle*, *cell adhesion*, *blood vessel development*, and *cell migration* in the time window between 37 and 48 hours.

Four Rho GTPases were up-regulated in this time window. Two of these (RHOC and RND3) were among the Rho GTPases with a strong grade IV specific connectivity profile in multiple datasets (Fig 3D) and the remaining two (RHOBTB1 and RHOBTB2) were non-specific. Since GTPases and their potential transcriptional targets were all modulated at this specific time window we hypothesized that if a cause and effect relationship exists it should be within the time frame and resolution of our sampling (12 hours).

We therefore reverse engineered a static ARACNE mutual information network representing the neighbourhood of RND3, RHOC, RHOBTB1 and RHOBTB2 during the tumour implantation time course (S4A Fig). Remarkably, we found that the resulting network shared many properties with the grade IV network derived from the clinical study (Fig 3). First of all, the GTPases which we predicted to be grade IV specific had a markedly larger number of connections. Secondly, we discovered that RND3 was the most connected gene (S4B Fig) at a high level of stringency (p < 10^−8^). Furthermore, we could verify a high degree of functional overlap between genes connected to RND3 in the static CAM network and the grade IV network inferred from the clinical samples (Fig 3E; S1 Table), making this gene an ideal candidate for further analysis.

We then sought to experimentally define the transcriptional response linked to RND3 and compare this with its predicted targets in the inferred grade IV networks. We therefore used RNA interference to knock-down expression of RND3 by siRNA in U87 cells (siRND3) *in vitro* and performed an expression profiling analysis. Differential expression analysis revealed 2,606 genes up-regulated and 2,099 genes down-regulated compared to non-silencing controls (siControl/siRND3). A Gene Ontology analysis of these genes was consistent with the predictions made from the reverse engineered networks (Fig 3E; S1 Table). The full Gene Ontology analysis can be found in S5 Dataset. Functions related to tumour development including *inflammatory response*, *regulation of apoptosis*, *cell migration* and *cell cycle* were differentially regulated in directions consistent with the correlation patterns within primary glioma and U87 implantation networks. Additionally, we noticed that RND3 knockdown resulted in the transcriptional up-regulation of genes related to *DNA repair*, *histone modification*, *RNA splicing* and *transcription factor binding*, and down-regulation of genes in *cell communication*, *amino acid phosphorylation*, *cell growth* and *response to oestrogen* functional terms (S5 Fig). We also noticed that the transcription of key genes involved in inflammation, proliferation, angiogenesis and extracellular matrix remodelling (including MMP2, HIF1A, IL1B, IL1A, IL1R1, MMP2, VEGFA), processes vital to glioblastoma development, were down-regulated by RND3 knock-down (S6 Fig).

### RND3 is up-regulated in grade IV glioma

**Fig 4:**
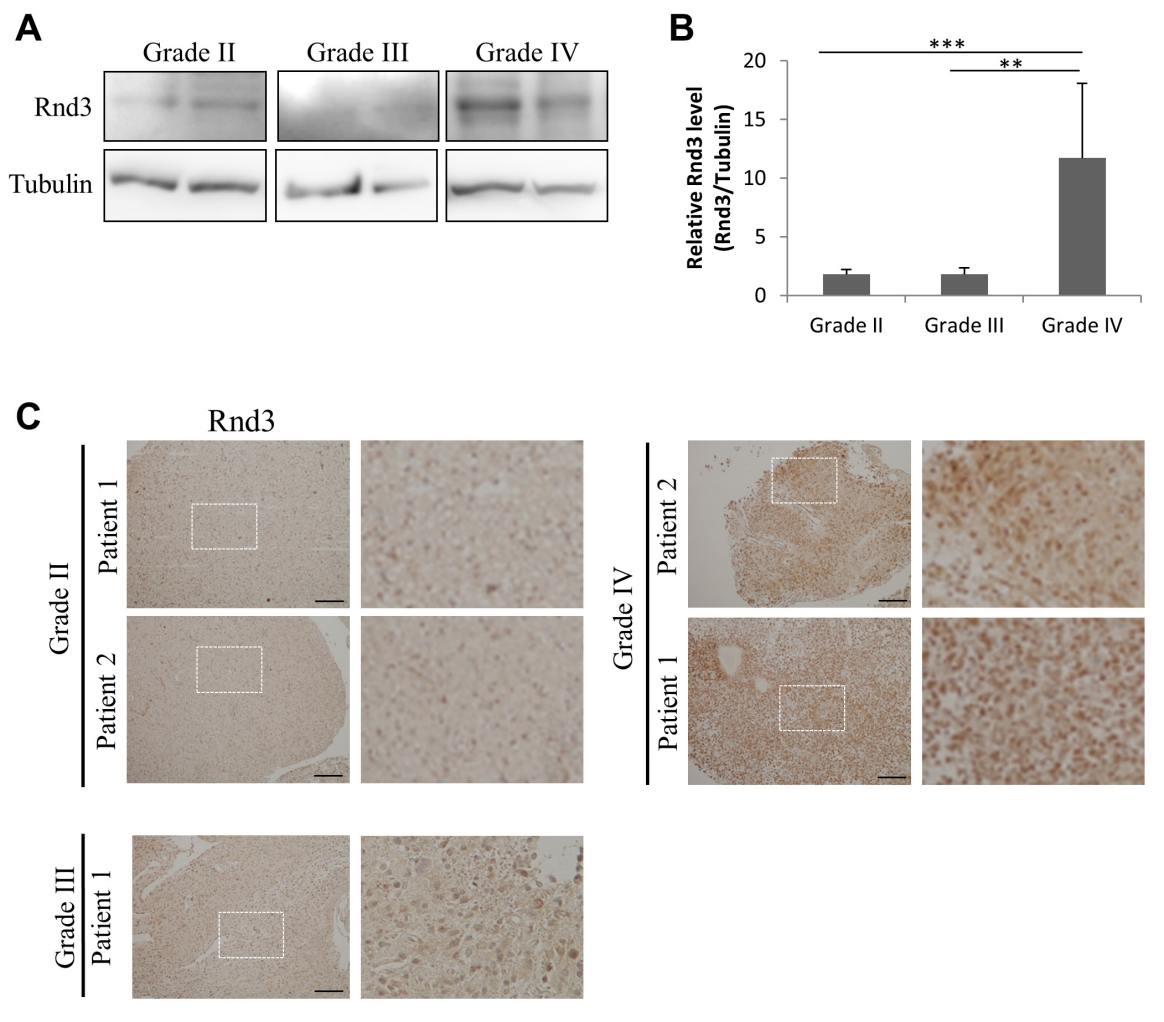
RND3 is up-regulated in grade IV glioma. A. RND3 expression determined by western blot in patient samples from different glioma grades as determined after anatomopathological analysis. B. Densitometric analysis of RND3 protein from western blot shown in (A) presenting the ratio of RND3 to tubulin. C. Immunohistochemical analysis of RND3 expression in grade II, III and grade IV patient tumors (*bar* 100 μm), magnified images are presented in left panels. Data is representative of 4 or more tumours. *** p < 0.001, ** p < 0.01, * p < 0.05, values +/- SEM.

RND3 and other Rho GTPases are transcriptionally regulated in high-grade glioma (see S2 Fig). However, the expression of RND3 protein in different glioma grades is unknown. We tested the expression of RND3 which on the basis of the numbers of inferred network connections we predicted to have differential activity across glioma grades. Remarkably, western blot analysis of grade II, III and IV human glioma samples confirmed that protein expression correlated with the transcriptional network connectivity. RND3 protein was significantly up-regulated in grade IV gliomas compared to both grade II and grade III (Fig 4A and 4B). We confirmed the difference in RND3 expression in tumour cells by immunohistochemistry analysis of grade II, III and IV gliomas (Fig 4C). RND3 was found in the cytoplasm of tumor cells but also in the nucleus.

We then set to characterise RND3 expression in relation to glioblastoma sub-types and genetic mutations in key disease genes. We discovered that RND3 is up-regulated up to 2-fold in GBM of the mesenchymal subtype with respect to the others (S13A Fig). Consistent with this observation, other markers of mesenchymal subtype (MET, TLR4, RELB, TNF receptor and CD44) were significantly down-regulated following RND3 knock down in glioblastoma cells (S13B Fig).

We then tested whether individual genetic markers (copy number variations and SNPs) may be able to explain the expression of RND3. Interestingly, we were able to explain up to 5% of variance in RND expression with individual copy number variations and individual gene expression measurements in the EGFR and CDKN2A genes. In addition, the expression of NF1 was also able to explain a small part of RND3 expression (S13C Fig). We also used a random forest regression approach to find combinations of genetic markers that could explain RND3 expression. The resulting models were able to explain 14% (model with only genetic mutations) and 21% (Model including both genetic mutations and gene expression) of the variance in RND3 expression (S13D and S13E Fig).

### Experimental validation *in vitro* confirms the role of RND3 in controlling glioma cell proliferation, apoptosis, cell migration and invasion

The observed transcriptional changes suggest alterations in cell proliferation, migration and cell cycle. In order to test whether the transcriptional signature truly reflects physiological changes we first used RNA interference and a panel of *in vitro* assays to test proliferation, invasion, migration and cell cycle.

**Fig 5:**
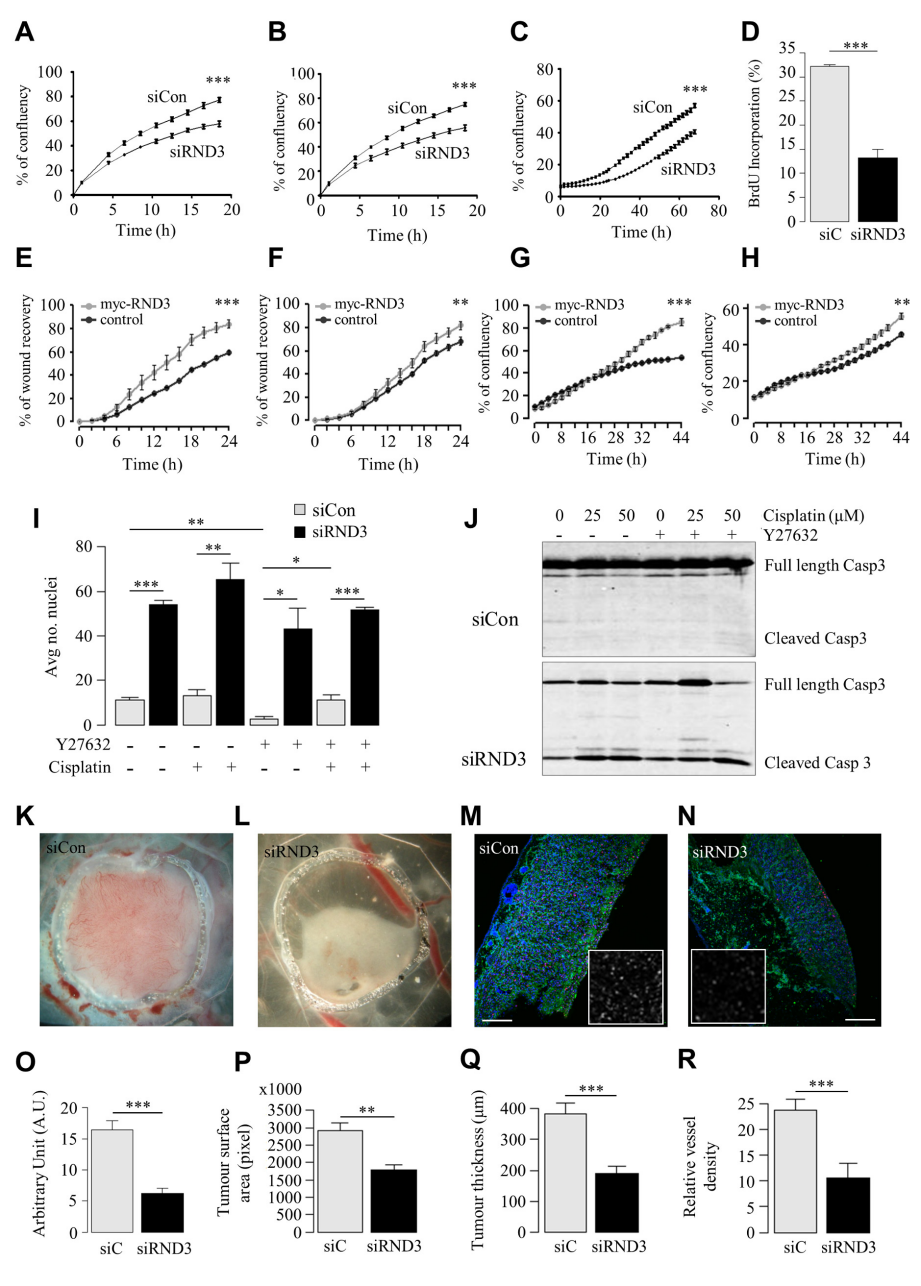
*In vitro* and *in vivo* RND3 silencing reduces cell proliferation and migration, induces apoptosis and reduces tumour mass. **A, B.** (**A**) Invasion and (**B**) migration of U87 siRND3 and siControl cells determined by scratch wound assay. **C.** Proliferation of U87 siRND3 and siControl cells determined by confluence measurements. **D**. Percentage of cells showing BrdU incorporation into DNA in U87 siRND3 and siControl cells. **E, F.** Migration of RND3 overexpressing (**E**) 1321N1 and (**F**) T98G cells and Turbo-GFP control cells determined by scratch wound assay. **G, H.** Proliferation of RND3 overexpressing (**G**) 132N1 and (**H**) T98 cells. **I.** The average number of condensed nuclei in U87 siRND3 and siControl cells treated with Y27632 and/or cisplatin prior to imaging. **J.** Levels of cleaved Caspase 3 in U87 siRND3 and siControl cells with/without Y27632 and cisplatin treatment were determined by western blot. **K, L.** Representative image of 5 day old tumours derived from (**K**) wild-type and (**L**) U87 siRND3 cells implanted on the chicken CAM. **M, N.** Representative image of Ki-67 expression in 5 day old tumours derived from (**M**) wild-type and (**N**) U87 siRND3 cells. Magnification x10, scale bar 200μm, inset magnification x40. **O-R.** Quantification of (**O**) Ki-67 expression, (**P**) tumour surface area, measured as tumour area in pixels, (**Q**) tumour thickness, (**R**) relative density of blood vessels in 5 day old tumours. **A-J.** Data is representative of 3 independent experiments. **K-R.** Data is representative of 4 or more tumours. *** p < 0.001, ** p < 0.01, * p < 0.05, values +/- SEM.

The results obtained were fully consistent with our predictions, suggesting that knock-down of RND3 induced an anti-tumour phenotype in U87 cells, which express high levels of RND3 (S7A Fig). Proliferation, migration and invasion were all significantly reduced in U87 RND3-depleted cells (Fig 5A–5C). Bromodeoxyuridine (BrdU) labelling of RND3-depleted cells revealed significantly reduced numbers of cycling cells (Fig 5D; siRND3 U87 18.8±0.55% cells; control 32.3±0.32% cells).

We then performed an over-expression experiment to test whether increased levels of RND3 may have the expected effect on proliferation and migration. Firstly, the expression of RND3 was determined in several cell lines from different glioma grades. As shown in patient samples, a grade II cell line, 1321N1, expressed low levels of RND3 (S7A Fig). Two grade IV cell lines were assessed, U87 and T98G cells. T98G cells expressed a much lower amount of RND3 than U87 cells (S7A Fig). A lentiviral expression of myc-RND3 was then used to over-express RND3 in both low-expressing cell (S7B Fig). Both cell line morphologies were changed by myc-RND3 overexpression and this was validated by RND3-GFP over-expression (S7C Fig). F-actin staining showed formation of a large lamellipodia at the front of the cells and a decrease of cell volume.

This overexpression impacted both cell lines in a manner fully consistent with the RNA interference assays. Both proliferation and migration (Fig 5E–5H) were significantly increased in both 1321N1 and T98G cells. These results confirmed the importance of RND3 in cell aggressiveness behaviours.

We then validated the hypothesis that RND3 expression is linked to apoptosis. RND3-depleted cells showed an increase in cell death evidenced by a dramatic increase in both condensed nuclei (Fig 5I) and cleavage of caspase 3 (Fig 5J). Use of the small molecule inhibitor Y-27632 to inhibit ROCK activity did not significantly affect cell death in RND3-depleted cells suggesting that the effects of depleting RND3 were not mediated via ROCK1, which interacts with RND3 and is essential for its canonical function controlling cytoskeleton remodelling [23].

### RND3 knockdown blocks *in vivo* tumour expansion

Encouraged by the *in vitro* analysis we performed an *in vivo* implantation of U87 siRND3 cells in the chicken egg CAM. Phenotypic characterization of the resulting tumours was again fully consistent with the results of the *in vitro* analysis. Visual inspection showed that tumours appeared dramatically reduced in size 48 hours after implantation (Fig 5K and 5L). This was accompanied by a substantial reduction in proliferating cells shown by a 62% reduction in Ki-67 expression (Fig 5M–5O). Further quantification of the tumour surface area in a horizontal section confirmed an average 40% reduction in tumour expansion (Fig 5P; siRND3 U87 1.8x10^6^ pixels ±1.7x10^5^; control U87 2.9x10^6^ pixels ±2.3x10^5^). We also quantified tumour thickness after immunostaining which demonstrated a reduction of 50% (Fig 5Q; siRND3 U87 188.8μm ± 23.2; control U87 382.3μm±37.3). Consistent with the reduced expression of VEGFA mRNA in siRND3 transfected U87 cells, blood vessel density in siRND3 tumours was significantly lower compared to control tumours (Fig 5R; siRND3 oligo B U87 10.6±2.79; control U87 23.8±2.13), suggesting a disrupted angiogenesis process. Additional histological analysis of tumours revealed reduced Vimentin expression in line with the reduced tumour mass (S8 Fig). Pericyte coverage of blood vessels within the tumour, detectable by Desmin staining, was increased in siRND3 tumours (S8 Fig). Taken together, this supports a critical role of RND3 in tumour expansion by modulating angiogenesis, cell migration, invasion, apoptosis and cell cycle dynamics.

### A novel multi-level data integration algorithm identifies a potential mechanism for RND3-dependent cell cycle control

Some of the pro-tumour effects of RND3 (migration and invasion) are likely to be a direct consequence of its known role in cytoskeleton remodelling [24]. However, the mechanisms behind control of cell proliferation and apoptosis are less easy to interpret, particularly considering that these are ROCK1 independent (Fig 5I and 5J). Therefore, in order to further explore RND3-dependent growth and survival, we designed an unbiased, open-ended approach based on the de-novo identification of RND3 interacting proteins followed by computational analysis.

Co-immunoprecipitation of RND3 flag tagged protein and subsequent mass spectrometry analysis identified 205 putative interactors of RND3. Functional profiling of the interactors revealed primarily nuclear associated proteins involved in *regulation of translation*, *the nuclear lumen*, *intracellular transport* and *cell division* (Table 1).

**Table 1:** Gene Ontology categories of 205 putative RND3 binding partners. Gene Ontology terms in bold were significantly enriched (false discovery rate < 10%).

Category	Term
GOCC	**Nuclear lumen (55)**
GOMF	**Nucleotide binding (62)**
GOMF	**ATPase activity (23)**
GOBP	**Intracellular transport (22), protein transport (19)**
GOBP	**Regulation of translation (9)**
GOBP	**DNA repair (12)**
GOCC	**Chromosome (18)**
GOCC	**Mitochondrial envelope (13)**
GOBP	**Cell division (10)**, Cell cycle (15)

In order to identify a specific mechanism that may explain RND3 tumour growth we developed a novel network modularization approach designed to identify a sub-network of the human interactome enriched with proteins interacting with RND3 and at the same time highly correlated in the CAM expression profiling time course.

**Fig 6:**
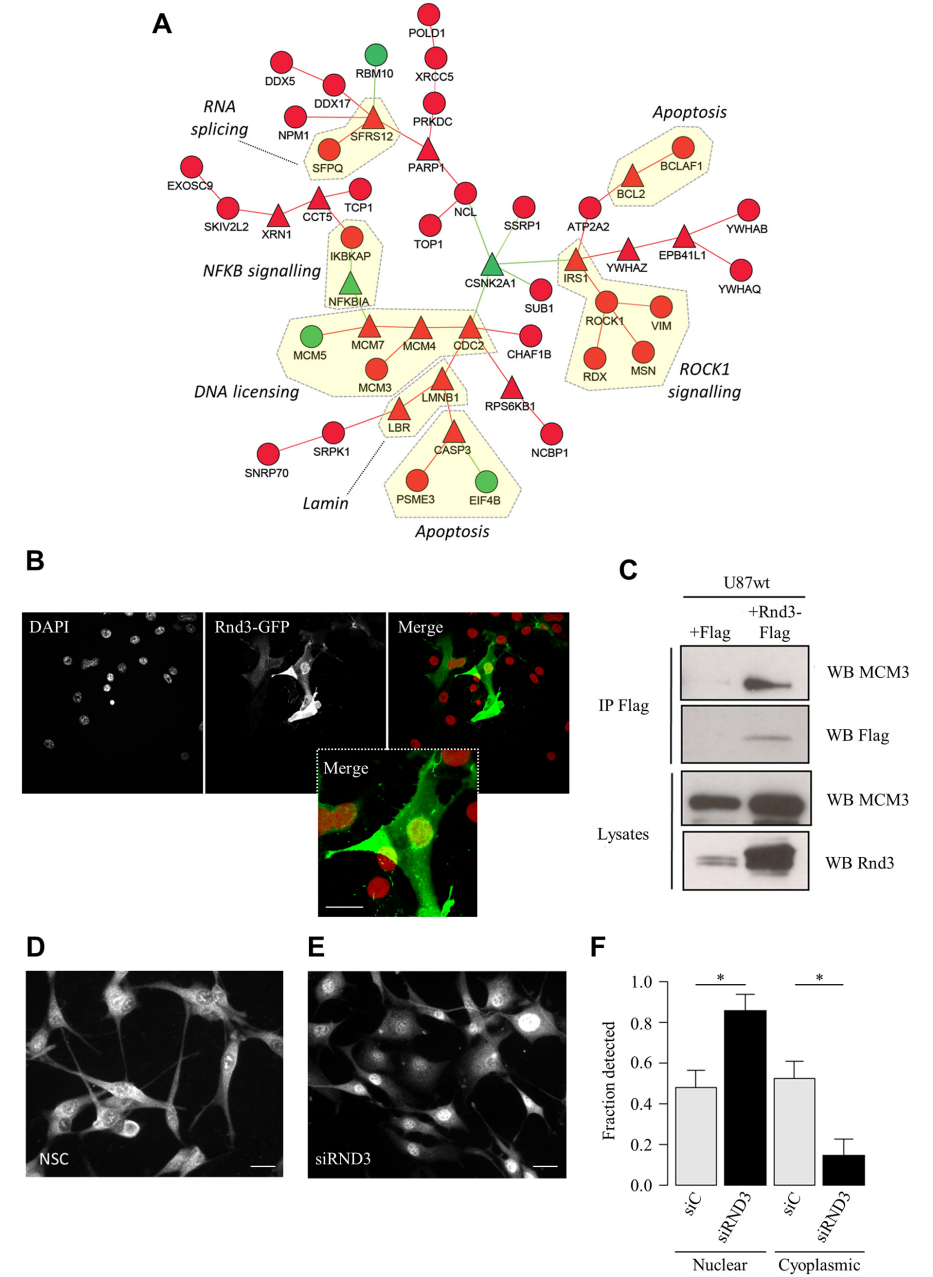
Reverse engineering networks from integrated datasets reveals a novel role of RND3 in cell cycle control. **A.** Protein-protein interaction network representing a module of RND3 interactors that are co-expressed during tumour development in the CAM. **B.** Confocal microscopy of U87 cells expressing RND3-GFP with DAPI stain (red). Scale bar: 10μm. **C.** Immunoprecipitation using anti-Flag antibody in U87 cells transfected with RND3-Flag or Flag. Western blot analyses used the indicated antibodies. **D, E.** Immunostaining of MCM3 in (**D**) U87 siControl cells and (**E**) U87 siRND3 cells. Scale bar: 10μm. **F.** Quantification of proportion of nuclear and cytoplasmic MCM3 detected by western blot in U87 siRND3 and siControl cells. Data is representative of 3 replicate experiments. *** p < 0.001, ** p < 0.01, * p < 0.05, values +/- SEM.

The algorithm (see S1 Text for details of the development and validation of the algorithm) identified a significant sub-network (p<10^−16^) containing 49 genes (Fig 6A). This network included genes known to be involved in Rho-mediated cytoskeletal remodelling (ROCK1, Vimentin, Moesin, Radixin) as well as components of NFKB signalling (IKBKAP, NFKBIA), apoptosis (Caspase 3, PSME3) and, interestingly, 4 nuclear proteins involved in DNA licensing (MCM3, MCM4, MCM5, MCM7) as well as the important cell cycle regulator CDC2. During the cell cycle RND3 expression has been shown to increase during G1 followed by a rapid decrease at S phase [25]. Taken together, this raised the hypothesis that RND3 may be associated to nuclear proteins and that this may be part of the mechanism regulating cell cycle. Consistent with recent reports [26] [27] and the immunohistochemistry analysis (Fig 4C) we demonstrated by confocal imaging of U87 cells that RND3 can localise to the nucleus (Fig 6B) and that it is detectable by western blot in nuclear fractions (S11 Fig). Complementing evidence of the nuclear occupancy of RND3, we performed Fluorescence recovery after photo-bleaching (FRAP) experiments (S12 Fig), showing motile RND3-GFP is able to partially recover into bleached regions in both the nuclear and cytoplasmic compartments. We hypothesised that RND3 might be controlling the cellular localisation of the nuclear DNA licensing factors and consistent with this we verified that MCM3 could be co-immunoprecipitated with RND3 (Fig 6C) in U87 cells. A previous report has suggested that high levels of MCM3 protein in the nucleus may result in cell cycle arrest [28]. We observed that depletion of RND3 leads to nuclear accumulation of MCM3 (Fig 6D–6F). Taken together, this suggests a novel role for RND3 in controlling cell cycle by modulating the localisation of the DNA licencing protein MCM3.

### Copy number variation (CNV) and expression of RND3 is predictive of clinical outcome

Having shown that RND3 plays a role in the development of GBM, we asked whether its expression could be influenced by genetic mutations such as copy number variation of the RND3 locus, and if both expression and CNV may be predictive of patient survival.

**Fig 7:**
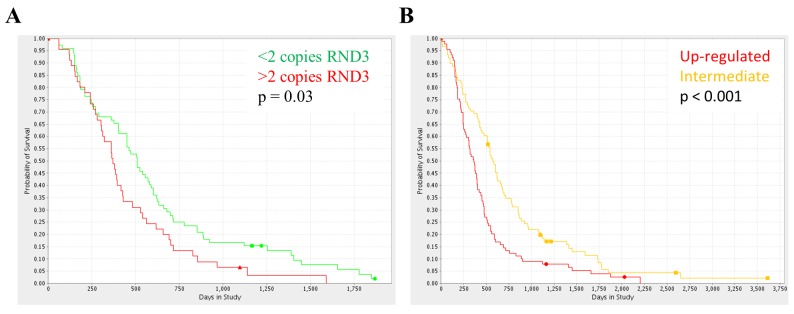
RND3 expression and copy number variation is a significant risk factor for glioma patients. **A.** The Kaplan-Meier survival curves for glioma patients with amplification (red, n = 45) or deletion (green, n = 72) of the RND3 locus, determined from SNP frequency data within the REMBRANDT database, log rank p value = 0.0301. **B.** The Kaplan-Meier survival curves for glioma patients with RND3 overexpression (red, n = 89) or comparable expression (orange, n = 88) versus non-tumour samples, log rank p value = 0.0006. All analysis performed using the online REMBRANDT database tools.

We first approached this question using data available within the REMBRANT database of functional genomics data [29]. We focused on GBM patients and found that patients with an increased RND3 copy number showed significantly lower probability of survival compared to patients with a normal or reduced RND3 copy number (Fig 7A). Using the same database we then could verify that RND3 gene expression was also predictive of survival and that RND3 CNV and expression were positively correlated (Fig 7B).

Next, in order to validate this initial finding, we performed survival analysis using the Cancer Genome Atlas (TCGA) database of GBM expression and copy number. Consistent with the survival analysis performed using REMBRANDT, both RND3 expression and CNV were predictive of survival (S9 Fig).

Taken together these finding suggest that genetic mutations can trigger increased expression of RND3 and that this correlates with clinical outcome.

## Discussion

The importance of our work is two-fold. Firstly, we demonstration that computational integration of multiple data sources is an effective strategy to unravel the structure of biological networks underlying the development of human glioma. Secondly, our models have highlighted that low and high-grade gliomas are characterised by a switch in the activity of two different sets of Rho GTPases, among which RND3 is a key regulator of tumour proliferation, migration, apoptosis and invasion. Furthermore, RND3 CNV correlates to its expression and is predictive of survival, suggesting that changes in the activity of this particular Rho GTPase could be an early event associated to transformation and tumour progression.

### The role of Rho GTPases in glioma

An important property that emerged from our inferred networks is the existence of two sets of Rho GTPases, which may target functionally different molecular networks and explain the more aggressive nature of high-grade Gliomas.

Overall, this hypothesis is consistent with the literature. Among the seven Rho GTPases that are linked to grade II networks we could find little evidence in the literature for a mechanistic linkage with tumour transformation or progression. On the contrary, we could find evidence of their involvement in normal tissue functions such as migration of neuronal shape change during brain development (RND2)[30], regulation of actin cytoskeleton involved in vesicle trafficking (RHOD, RHOF) [31] [32], sub-cellular trafficking of growth factor receptors in normal cells (RHOB) [33] [34], and protein degradation (RHOBTB3, CHP) [35] [36]. Among these, only RHOB has been firmly linked to high-grade glioma albeit with contrasting results. Repression of RHOB has been shown to increase motility and invasion in glioblastoma cells [37]. However, it has recently been shown to support glioblastoma tumorigenesis [38].

On the other hand, 6/7 of the Rho GTPases linked to high-grade glioma in our model has previously been shown to play a role in GBM. This includes effects on cell invasion (RAC1 [39], RAC2 [40], RAC3 [39], RHOA [41], RHOC [42]), focal adhesion formation (RHOA [43]), stemness of glioma precursor cells (RAC1 [44]), cell proliferation (RAC1 [45], RND3 [46]) and cell cycle (RND3 [25]).

### RND3, a pro-tumour gene in glioblastoma

Our work shows that down-regulation or overexpression of RND3 supports its role as a pro-tumour gene in glioblastoma controlling proliferation, migration and invasion using different glioma cell lines. Inactivation of RND3 also alters survival at both the level of cell cycle regulation and induction of apoptosis. Importantly, its expression appears to be regulated by genetic mutations such as CNV rather than as a secondary event down-stream of other cancer signalling pathways. Our work identifying RND3 as a pro-tumour gene is consistent with data from endometroid adenocarcinoma cells where RND3 is described as a p53 inducible pro-tumour gene, promoting proliferation and survival of cells following DNA damage [47]. However, its role in other types of cancer may be different. RND3 is under-expressed in prostate cancer and induces apoptosis and cell cycle arrest [48].

In addition, a previous study in glioma positioned RND3 as an anti-tumour gene, decreasing proliferation and inducing apoptosis in U87 cells [46]. However, these data were based on the overexpression of RND3 in a cell type that already expresses high levels of RND3 (S7A Fig), leading to a non-physiological situation. It is also possible that RND3 has a bell-shaped activity profile, where inhibition is seen when it is absent or low and when it is highly overexpressed such as after transfection into cells.

The strength of our approach, in contrast to many of these previous studies, relies in the fact that a systems biology approach that combines clinical and experimental datasets has been used in our study. This provides us with an array of concording results that clearly point to a pro-tumour role of RND3 in glioblastoma.

### Towards predictive modelling of glioma development

In this article, we have shown that by combining advanced network biology approaches with the right experimental models, we are able to reveal novel regulatory circuits controlling multiple hallmarks of cancer. However, these findings should stimulate several research directions. Firstly, while we now better understand the role and clinical relevance of RND3 in human Glioma, we still do not have a mechanistic model explaining the switch between networks of normal and abnormal regulatory Rho GTPases, which we hypothesise drives the establishment of a progressively more severe cancer phenotype, presumably at the expenses of normal glial function. In this context, we have identified MCM3, a DNA licencing factor, as a new interacting partner. This interaction may participate, in addition to its known modulatory activity on RhoA activity, to the biological effects triggered by RND3 in glioma cells. The specific role of the RND3-MCM3 interaction is at present not established. One may speculate that it is involved in nuclear-cytoplasmic shuttling. Establishing a complete mechanistic model will involve extensive experimental analysis of the role of each regulator and, eventually, the development of a mathematical model to simulate glioma progression. Ultimately, the integration of molecular, phenotypic and clinical endpoints within a computational model will provide a new set of investigative and predictive tools to support clinical decision-making.

## Methods

### Ethics statement

Human tumour samples are provided by Rolf Bjerkvig (University Bergen, Norway). He has ethical permission to store biopsy specimens from human patients in a biobank, as well as corresponding xenografts in animals. Regional ethical approval number: 013.09. The tissue collected is anonymized at the department of Neurosurgery at Haukeland University Hospital. Animal experiments: the animal experiments are conducted in the Animalerie Mutualisée, Bordeaux, France. The number of authorisation is B33-522-22 and was obtained February 28 2012. The approval for experimentation has been obtained from the University of Bordeaux ethical committee (approval number R-45GRETA-F1-10).

### Identification of genes differentially expressed in low and high-grade glioma

Differentially expressed genes between grade II (n = 45) and grade IV (n = 81) gliomas were identified using an existing microarray study by Sun *et al* [49]. Raw data were downloaded from the GEO database (accession GSE4290) and normalized using the Robust Multiarray Average algorithm (RMA) [50]. Statistical significance was determined using a t-test followed by correction for multiple comparisons using the Benjamini-Hochberg method to estimate the false discovery rate (FDR) [51]. Genes with a log_2_ fold change greater than 1.5 and an FDR < 1% were selected. This transcriptional signature was compared to a transcriptional signature from a similar study comparing grade II (n = 50) and grade IV (n = 24) glioma (accession GSE52009) for validation purposes. Both transcriptional signatures showed a very high level of similarity (FDR < 1%) when compared using Gene Set Enrichment Analysis [52] (S1 Fig).

### Modularization of the human protein interactome representing tumour stage transition

In order to achieve this goal we first subset the human protein interactome by selecting proteins encoded by genes differentially expressed between grade II and grade IV glioma samples. The interactions between this subset of proteins were identified using the Michigan Molecular Interaction (MiMI) protein interactions databases [53], which merged and integrated a number of protein interactions databases such as BioGRID [54] and HPRD [55]. This protein-protein interaction (PPI) network consisted of 1423 nodes and 18,681 edges. Hubs were identified within the network by calculating the percolation score [56] for each node, in order to identify potentially important proteins. There were 93 hub nodes with a percolation score greater than 2 standard deviations above the mean. The complete glioma stage network was constructed by selecting the first neighbours of these network hubs, resulting in 682 nodes and 2472 edges. The network was modularized using the GLaY algorithm for community detection [57].

### The chorioallantoic membrane (CAM) glioblastoma implantation model

U87 glioblastoma cells were maintained in DMEM, 10% FBS, antibiotics and L-glutamine. U87 cell pellets were deposited on the chicken egg CAM at developmental day 10 as previously described [16]. For the transcriptomics time-course, tumours were dissected from the CAM and snap frozen every 12 hours following implantation at developmental day 10 until developmental day 15 (10 time points). To assess the effect of RND3 silencing on tumour growth on the CAM, U87 cells were transfected with siRNA for RND3 or non-silencing control siRNA before implantation. Tumours were imaged every day and grown for 5 days after deposition on the CAM. The tumour was then dissected and mounted in OCT and processed for measurement and immunohistochemistry.

### Reverse engineering transcriptional networks in glioma

Low and high-grade glioma transcriptional networks were generated from grade II and grade IV samples from the *Sun et al* dataset (GSE4290) [49] and Cancer Genome Atlas datasets (TCGA) (http://cancergenome.nih.gov/). Normalised, batch corrected TCGA datasets were downloaded from the MD Anderson MBatch (http://bioinformatics.mdanderson.org/tcgambatch/) website. Mutual information networks were inferred using the ARACNE method [58] with the Rho GTPases set as hub genes. Significant gene-gene interactions were defined using a p-value cut-off of 1x10^-5^.

A dynamic model of gene expression in the developing tumour during the first 5 days following U87 deposition on the CAM has been inferred from our microarray data using a custom bioinformatics pipeline based on time-delay correlation. In order to reduce the complexity of the transcriptional response we generated 14 gene clusters with distinct expression profiles using the HOPACH algorithm [59]. The clusters were categorized into rapid (1–12hr), intermediate (13–24hr) or delayed-responders (37–48hr) to implantation (no clusters fell into the 25–36hr category) according to the time point at which the expression profile was altered by 50% of the dynamic range (S3 Fig). In order to increase the number of data points available for correlation analysis we first identified the median expression profile of each cluster and then applied a polynomial interpolation algorithm to generate 100 data points. The correlation matrix between the interpolated expression profiles was calculated using Spearman’s Ranking Coefficient with the addition of a time-delay procedure. Interpolated expression profiles were considered correlated with a time-delay if a shift of one expression profile by 13–24 hours (1–2 time points) improved the correlation value. The time-delay procedure identified the maximum correlation value within this time-delay window. Clusters with a highly stringent correlation value of >0.9 were considered significantly correlated.

The static transcriptional networks of genes highly correlated to expression of Rho GTPases in the CAM tumour implantation were identified using the ARACNE method [58]. A range of high stringency p value thresholds (p < 10^−6^, p < 10^−7^, p < 10^−8^) were applied in order to identify the most highly connected GTPases independent of statistical confidence.

### RND3 siRNA (siRND3) knock-down

To knockdown expression of RND3 U87 cells were transiently transfected with two custom siRNA oligos (9 pmol) supplied by Dharmacon (oligo A 5’-AUAGUAGAGCUCUCCAAUCA-3’ or oligo B 5’-CAAACAGAUUGGAGCAGCU-3’) using Lipofectamine RNAiMAX (Invitrogen) as described elsewhere [60]. Control, non-silencing, oligos were purchased from Qiagen. Knock-down was confirmed to last up to 96 hours by western-blot analysis (S10 Fig).

### Western blot analysis

Protein were separated by SDS-PAGE and then transferred to nitrocellulose membranes using previously described methods [61]. Western blotting was performed using antibodies against cleaved Caspase 3 (Cell Signalling Technology), RND3 (Millipore), MCM3 (Abcam), Lamin A/C (Santa Cruz Biotechnology), α-tubulin (Sigma) and Flag (Sigma M2). The western blot bands were quantified using ImageJ Software.

### Microarray analysis of implanted tumours and U87 cells

RNA was extracted from U87 cells and snap frozen tumours using RNeasy columns (Qiagen, UK). RNA purity was assessed using a NanoDrop spectrophotometer and each sample had a 260/280 ratio of 1.8–2.1. RNA was reverse transcribed and the cDNA was labelled with fluorescent Cy3 dye using the Agilent Low-input Quick Amp Kit (Agilent, UK). cRNA was purified using RNeasy columns (Qiagen, UK) and hybridised overnight to Agilent Human 8x60k Whole Genome or Agilent Chicken V1 Whole Genome microarrays according to the manufacturer’s protocol. Microarrays were scanned using an Agilent SureScan microarray scanner and processed using Agilent Feature Extraction software. Data was normalised using quantile normalisation.

In order to remove probes from the analysis that could potentially hybridise to both chicken and human cRNA we performed a separate microarray analysis. We created separate pools of RNA from CAM and U87 cells to create chicken and human reference samples. The chicken and human reference RNA was then hybridised to both human and chicken whole genome microarrays. RNA extraction, generation of fluorescently labelled cRNA, microarray hybridisation and scanning protocols were identical to those used for the implanted tumour tissue. After subtraction of the background signal, the relative contribution of the human cRNA to the total fluorescence observed for each probe on the chicken array was calculated, and vice versa. Any probe for which the cross-hybridisation of cRNA from the other species resulted in a fluorescent signal > 64 or the relative contribution to the total signal was greater than 15% was removed. 5,272 probes were removed from the chicken dataset and 9,128 probes were removed from the human dataset.

Genes differentially expressed during the time course of U87 implantation on the CAM were detected using a two-step method. First, genes with a minimum fold change of 1 in log_2_ scale were selected, then noisy genes were removed using the BETR [62] algorithm with α = 0.001.

Genes differentially expressed in U87 cells in response to siRND3 treatment were detected using the SAM method [63]. Genes with a false discovery rate of 5% or lower were deemed significant.

### Development of a novel network modularisation algorithm and its application to define the RND3 interactome

We developed a procedure derived from the work of Dittrich *et al* [64], which uses the prize-collecting Steiner tree framework to identify network modules in protein-protein interaction data. However, our novel procedure is able to integrate three independent sources of data including known protein-protein interactions, differential gene expression and correlation structure. This was applied to identify networks of RND3-interactors that are enriched with co-expressed genes linked to glioma grade and therefore potentially important for tumour progression. The development of the algorithm and application to simulated data is described in S1 Text.

### Cell proliferation, migration and invasion assays

For proliferation, migration and invasion assays U87 (5x10^4^ cells/well) cells were plated in 96-well plates. For siRNA proliferation experiments, siRND3 or siControl transfections were performed 24h before plating. 1x10^5^ or 5x10^5^ T98G or 1321N1 cells respectively were plated for proliferation and migration assays. Each cell line was infected with control GFP-Turbo or myc-RND3 lentiviral plasmids to a MOI of 10. IncuCyte technology (Essen Bioscience) was used to generate measurements of cell proliferation, migration and invasion over time. Growth curves (proliferation) were built from confluence measurements acquired during round-the-clock kinetic imaging. For invasion and migration assays cells were plated in 96-well ImageLock plates (Essen Bioscience). Wells were pre-coated for 6h with 50μg/ml of reduced matrigel (BD Biosciences). For invasion assay 150μg/ml of reduced matrigel was added on each well. At 90–100% of confluence the plates were scratched with a 96-Well WoundMaker (Essen Bioscience). Migration/invasion was detected by IncuCyte scanning one image per well, every two hours for 18 hours. The time-course of cell migration/invasion was quantified using percentage of scar recovery (cells migrated/invaded into the wound) at 2 h time intervals. Proliferation was measured by Bromodeoxyuridine (BrdU) labelling of U87 cells using the BrdU Labelling and Detection Kit I (Roche) according to the manufacturer’s instructions.

### Apoptosis assays

To induce apoptosis, U87 cells were treated with 5μM ROCK inhibitor Y-27632 and/or 50 μM cisplatin (cis-diammineplatinum(II)dichloride) for 16 hours and apoptosis assessed by detection of cleaved caspase 3 by western blot or by staining cells with DAPI (4',6-diamidino-2-phenylindole, Invitrogen) and counting the number of cells with condensed nuclei, as previously described [60].

### Immunohistochemistry

Tumour grafts grown on chicken egg CAM were excised, fixed with 4% paraformaldehyde for 5 minutes and processed for cryo-sectioning. Ten micrometre sections were placed on Super Frost slides and immunohistochemistry was performed directly after fixation of the tissue on the slide with 4% paraformaldehyde. For immunohistochemistry, we used the following primary antibodies: anti-human Vimentin (1:400; Santa Cruz), anti-human Ki-67 (1:200; Santa Cruz) and anti-Desmin (1:100; clone D33 from DAKO). Corresponding fluorescent secondary antibodies were from Molecular Probes (1:1,000, Invitrogen). Chick blood vessels were visualized by using fluorescein-coupled *Sambucus nigra* lectin-FITC (SNA-1 lectin, 1:100, Vector Laboratories). Cell nuclei were visualized by DAPI (Invitrogen). Fluorescent labelling was viewed by confocal microscopy (Nikon). Quantification of staining was performed using ImageJ software.

Paraffin-embedded formalin-fixed glioma tissue sections were deparaffinized and heated at 99°C for 20 min in 10 mM citrate buffer at pH 6.0 or incubated with proteinase K diluted in 0.05 M Tris—Cl, pH 7.5 at 37°C for 10 min. The sections were incubated with the following primary antibodies: anti-RND3 (Abcam ab79999, 1/100). Primary antibodies were incubated overnight at 4°C. Detection was performed using a biotinylated secondary antibody (Vector Laboratories) amplified with Vectastain ABC Reagent (Vector). Sections were developed using 3′3-diaminobenzidine (DAB, DAKO), following the manufacturer’s instructions. The immunohistochemical stainings were analyzed and pictures were taken with a Nikon light microscope (Nikon Eclipse E600, Melville, NY, USA) using Nikon imaging software (Nikon NIS Elements v 4.11).

Imaging of MCM3 localisation was performed on U87 cells transfected with siRND3 or non-silencing control oligos. PFA (4%) fixed cells were stained for MCM3 (1:200, Abcam) and fluorescent labelling was viewed using a Nikon Eclipse Ti system.

### Identification and validation of RND3 binding proteins

HEK293T or U87 cells were transfected with pCMV-Flag-RhoE (RND3) or pCMV-Flag as a control as previously described [23]. To discover putative RND3 binding proteins cell lysates were immunoprecipitated using anti-Flag conjugated beads (Sigma). After SDS-PAGE and staining with coomassie blue the gel was cut into 7 equal fragments and subjected to in-gel trypsin digestion (along with matching gel slices from empty vector control) and analysed by mass spectrometry.

Co-immunoprecipitation of Flag-RND3 with Mcm3 was performed on U87 cells. Cells were washed with ice-cold serum-free medium and lysed on ice in buffer containing 20 mM Tris-HCl (pH 7.4), 150 mM NaCl, 1 mM EGTA (pH 8.0), 1 mM EDTA (pH 8.0), 2.5 mM pyrophosphate, 1 mM β-glycerophosphate, 1% Triton X-100 containing freshly added protease, and phosphatase inhibitor cocktail tablets (Roche). Lysates were clarified by centrifugation at 4°C, and the protein concentrations were determined by using Bio-Rad protein assay reagent (Bio-Rad Laboratories). For immunoprecipitation analyses, aliquots of cellular lysates were incubated with 2 μg of monoclonal anti-Flag (Sigma M2) for 1 h at 4°C. Immunocomplexes were collected on protein G-Sepharose beads (Sigma). The beads were washed three times with lysis buffer then boiled for 5 min in Laemmli sample buffer.

### Mass spectrometry

HEK293T cells were used for mass spectrometry analysis. UltiMate 3000 HPLC series (Dionex, Sunnyvale, CA USA) was used for peptide concentration and separation. Samples were trapped on uPrecolumn Cartridge, Acclaim PepMap 100 C18, 5 um, 100A 300μm i.d. x 5mm (Dionex, Sunnyvale, CA USA) and separated in Nano Series^TM^ Standard Columns 75 μm i.d. x 15 cm, packed with C18 PepMap100, 3 μm, 100Å (Dionex, Sunnyvale, CA USA). The gradient used was from 3.2% to 44% solvent B (0.1% formic acid in acetonitrile) for 30 min. Peptides were eluted directly (~ 300 nL min^-1^) via a Triversa Nanomate nanospray source (Advion Biosciences, NY) into a LTQ Orbitrap Velos ETD mass spectrometer (ThermoFisher Scientific, Germany). The data-dependent scanning acquisition in positive ion mode was controlled by Xcalibur 2.7 software. The mass spectrometer alternated between a full FT-MS scan (m/z 380–1,600) and subsequent collision-induced dissociation (CID) MS/MS scans of the 7 most abundant ions. Survey scans were acquired in the Orbitrap with a resolution of 30,000 at m/z 400 and automatic gain control (AGC) 1x10^6^. Precursor ions were isolated and subjected to CID in the linear ion trap with AGC 1x10^5^. Collision activation for the experiment was performed in the linear trap using helium gas at normalized collision energy to precursor m/z of 35% and activation Q 0.25. The width of the precursor isolation window was 2 m/z and only multiply-charged precursor ions were selected for MS/MS. MS/MS scans were searched against NCBI database using Mascot algorithm in Proteome Discoverer 1.1 software (Thermo Fisher Scientific). Variable modifications were deamidation (N and Q), oxidation (M) and phosphorylation (S, T and Y). The precursor mass tolerance was 10 ppm and the MS/MS mass tolerance was 0.8Da. Two missed cleavage was allowed and were accepted as a real hit protein with at least two high confidence peptides.

### Preparation of nuclear and cytoplasmic fractions

To prepare nuclear and cytoplasmic fractions trypsinised cells (3x10^6^) were washed in ice cold PBS and incubated in 500μl RSB (10mM Tris pH 7.4, 5mM MgCl2, 10mM KCl) containing 0.5% v/v NP40 and protease inhibitors for 5 min on ice before being centrifuged at 500g for 5 minutes. 150μl of the supernatant (cytoplasmic fraction) was removed and 30μl of 6x protein sample buffer added. The pelleted nuclei were resuspended in 1ml of RSB and centrifuged for 5 minutes at 500g. This was repeated and the pellet re-suspended in 150μl of protein sample buffer. To confirm clear separation of nuclear and cytoplasmic fractions lysates were analysed by western blot with Lamin A/C (nuclear marker) and α-tubulin (cytoplasmic marker).

### Single cell imaging

Single cell imaging of RND3 localisation was performed using U87 cells transfected with RND3-GFP (Addgene #23229) alone (with DAPI stain) or RND3-GFP and H2B-mcherry (Addgene #21044) plasmids. Transfection involved 24h incubation with a mixture of TransIT-LT-1 transfection reagent (Mirus BIO) in a ratio of 3:1 with plasmid DNA. Confocal imaging was carried out on a Zeiss LSM510 microscope using either 20x Fluar 0.8 NA or 63x Planapochromat 1.4 NA objectives at a temperature of 37°C, 5% CO2 and humidified atmosphere. Z-stack images were taken at sequential 1um depth slices. Fluorescence recovery after photo-bleaching (FRAP) experiments involved similarly-sized regions of the cytoplasm and nuclear compartments exposed to 20 iterations of a 488nm argon-ion laser set to 100% power.

FRAP and Z-stack live cell imaging was carried out at the Centre for Cell Imaging, IIB, University of Liverpool, UK.

### Linking RND3 to GBM subtypes

To investigate if expression of RND3 is dependent on GBM subtype [8], genes with known signatures and genetic mutations influencing the subtype were compared with RND3. The eight genes included: NF1, PDGFRA, IDH1, EGFR, TP53, FIP1L1 (FIP1L1 can become a fusion protein with PDGFRA), PTEN and CDKN2A. Normalized gene expression, scaled CNV and somatic mutation data free from batch effects was downloaded from the MD Anderson Bioinformatics Cancer Genome Atlas MBatch resource (bioinformatics.mdanderson.org/tcgambatch). A regression analysis was performed using either univariate regression or the ensemble classification and regression algorithm Random Forest (RF), where RND3 was used as the dependent variable and the 8 gene CNV and expression data as the predictor variables. The most influential predictor variables and the % variability of RND3 expression explained by the RF models are reported.

### Survival analysis

The effects of alterations in expression and copy number variation of Rho GTPases on glioblastoma patient survival was assessed using the REMBRANDT [29] and TCGA databases. The online tools available on the REMBRANDT website (www.caintegrator.nci.nih.gov/rembrandt) were used to generate Kaplan-Maier survival curves and log rank p values. Similarly, the TCGA data was analysed by first calculating ranks for each patient according to the expression or standardised CNV of RND3, and then finding the optimum partitioning of patients that maximises the significance of the Cox regression model. This was then used to generate Kaplan-Maier survival curves and log rank p values.

### Accession numbers

The following datasets are deposited within Gene Expression Omnibus: Implantation of U87 cells on the chicken CAM (GSE43674), RND3 silencing in U87 cells (GSE43812).

## Supporting Information

S1 Dataset Gene Ontology analysis of the genes found within network modules M1–M4. Gene lists have been split into those up-regulated or down-regulated in grade 4 glioma compared to grade 2 glioma. Significantly enriched Gene Ontology terms were selected using a 10% False Discovery Rate cut-off.
(XLSX)

S2 Dataset Rho GTPase gene connectivity in networks derived from the Cancer Genome Atlas glioma grade II and IV datasets. Rho GTPase connectivity was assessed in multiple ARACNE networks at different significance levels for gene-gene connections (p-values < 1e^-5^ or 1e^-6^). The same threshold used for the original data was used for defining Rho GTPases with strong grade specific connectivity (the proportion of grade II connections / proportion grade IV connections > 0.4 or < -0.4).
(XLSX)

S3 Dataset Gene Ontology analysis of genes connected to Rho GTPases in the grade II and grade IV gene expression networks. The Gene Ontology analysis of the network neighbourhood of grade II specific Rho GTPases in grade II networks and the network neighbourhood of grade IV specific Rho GTPases in the grade IV networks are shown in separate worksheets. No filtering has been applied, all mapped Gene Ontology terms and the relevant genes are included.
(XLSX)

S4 Dataset Gene lists and Gene Ontology terms mapped to gene clusters in the high level map of transcriptional changes in the chicken CAM glioblastoma implantation model. Gene Ontology analysis has been performed on genes regulated at each level of the transcriptional map (1–13hrs up, 13–24hrs down, 13–24hrs up, 37–48hrs down, 37–48hrs up-regulated). All mapped Gene Ontology terms are included.
(XLSX)

S5 Dataset Gene Ontology analysis of genes up-regulated in U87 siRND3 cells compared to U87 siControl cells. All mapped Gene Ontology terms are included.
(XLSX)

S1 Fig A comparison of differentially expressed genes between grade II and grade IV glioma in two independent datasets. Enrichment score (ES) plots generated by comparing differentially expressed genes between grade II and grade IV glioma in GSE4290 and GSE52009 datasets using the Gene Set Enrichment Analysis software. False discovery rate <0.001% for both comparisons.
(TIF)

S2 Fig Rho GTPase gene expression in different glioma grades. The expression of Rho GTPases in the *Sun et al* dataset is represented as a colour heatmap. Gene expression is standardised by row for visualisation. Mean linear fold change values are indicated for each glioma grade comparison. Statistical significance is determined by t-test followed by Benjamini-Hochberg FDR correction.
(TIF)

S3 Fig Correlation network of gene expression changes in U87 derived tumours implanted on the chicken egg CAM. 14 distinct gene clusters are categorised as early (1–12hrs; top row), intermediate (13–24hrs; middle row) or delayed responders (37–48hrs; bottom row) according to the time point at which the gene expression is altered by 50% of the dynamic range of the cluster. The time window (pink bar) and exact position in the interpolated time series (dotted line within pink bar) at which the cluster has altered by 50% of the clusters dynamic range is shown in respect to the medoid expression profile of each cluster (black line) and the interpolated expression profile (red line). Significant time-delay correlations between expression profiles are shown as dotted lines between clusters. Genes within each group of clusters (early, intermediate, and delayed) were determined to be up or down-regulated according to the expression trend of the cluster. A representative selection of Gene Ontology terms enriched within up (red) and down-regulated (green) genes at each level are coloured accordingly (false discovery rate < 10%). ns: non-significant. Individual genes within each cluster are coloured according to function: Rho GTPase (blue), secreted factor (green) and known oncogene (red).
(TIF)

S4 Fig The network neighbourhood of Rho GTPases in the CAM implantation model reveal RND3 is a hub gene. **A.** The gene neighbourhood of Rho GTPases RHOBTB1, RHOBTB2, RND3 and RHOC in the CAM network (mutual information p value < 10^−6^). **B.** The size of the gene neighbourhood of RND3 and RHOC at a range of high statistical thresholds.
(TIF)

S5 Fig Functional analysis of genes differentially expressed in U87 cells following RND3 siRNA treatment. Heatmap representing expression profiles of differentially expressed genes between U87 siControl (n = 3) and U87 siRND3 (n = 3) cells. The number of genes matched to Gene Ontology terms within up and down-regulated genes are shown. Terms in bold are significantly enriched (false discovery rate < 10%). ns = non-significant.
(TIF)

S6 Fig Expression of key genes controlling angiogenesis, cell proliferation and extracellular matrix remodelling is reduced in U87 siRND3 cells. Heatmap representing expression profiles of a selection of differentially expressed genes related to extracellular processes.
(TIF)

S7 Fig Ectopic expression of RND3 in low expressing RND3 cells induces aggressive cell behaviors. **A.** Western blot analyses with RND3 and tubulin antibodies on U87, T98G and 1321N1 cells. **B.** Western blot analyses with RND3 and tubulin antibodies on myc-RND3 expressing T98G and 1321N1 cells. **C.** Ectopic expression of RND3-GFP or myc-RND3 in T98G and 1321N1 cells. RND3-GFP or myc-RND3 are represented in green and F-actin/DAPI stainings in red and blue respectively. Scale bars, 10 μm.
(TIF)

S8 Fig *In vivo* staining of Vimentin and Desmin after implantation of U87 siRND3 on the CAM. Biomicroscopic pictures from tumors grown on the CAM. **A-D.** Representative immunohistochemistry staining of vimentin (**A, B**) or desmin (**C, D**). Tumors derived from U87 siControl (**A, C**) or U87 siRND3 (**B, D**) cells. Magnifications: (**A, B**) x10; scale bar 200μm, (**C, D**) x20; scale bar 100μm.
(TIF)

S9 Fig RND3 is predictive of survival in TCGA datasets. Kaplan-Meier survival curves of glioma patients partitioned by lower or higher RND3 expression and CNV in TCGA datasets. (**A**) The higher RND3 expression (n = 70) group has significantly lower survival rates than the low RND3 expression group (n = 165), p = 0.012, HR = 1.1–2.2. (**B**) The higher RND3 CNV (n = 317) group has significantly lower survival than the low RND3 CNV group (n = 119), p = 0.011, HR = 1.1–1.7.
(TIF)

S10 Fig RND3 knockdown is maintained for 96 hours in U87 cells. RND3 expression determined by western blot in U87 cells 24, 48, 72 and 96 hours after transfection with RND3 siRNA.
(TIF)

S11 Fig Western blot showing expression of RND3 within nuclear fraction of U87 cells. **A.** Detection of RND3 in whole cell lysates, nuclear fraction and cytoplasmic fraction of U87 cells determined by western blot. Lamin A/C and α-tubulin are included as exclusively nuclear/cytoplasmic controls respectively. **B.** Quantification of the western blot analysis by densitometry.
(TIF)

S12 Fig RND3 is expressed within the nucleus. **A-B.**Fluorescence recovery after photo-bleaching (FRAP) time-course for U87 cells expressing RND3-GFP. Cells were bleached after indicated time point using 20 iterations of 488nm laser set to 100%. Same-sized regions were bleached in the (**A**) entire nucleus (n = 14 cells) and (**B**) fraction of the cytoplasm (n = 6 cells). Fluorescence recovery was monitored and data plotted normalised to pre-bleach fluorescence intensity ± standard deviation. **C**. Image series showing representative U87 cell expressing H2B-mcherry (red) and RND3-GFP (green), imaged in sequential 1um depth ‘slices’ through the cell.
(TIF)

S13 Fig RND3 expression in Glioma subtypes. **A**. RND3 gene expression in patients with glioblastoma subtypes defined by Verhaak et al, 2010. Data consists of glioblastoma patients from the Cancer Genome Atlas database. **B**. Change in expression of genes characteristic of the mesenchymal subtype in U87 cells after RND3 silencing. **C**. Significance and percentage of variance in RND3 expression explained by univariate regression analysis using CNV or mRNA levels of frequently mutated genes in GBM. The top 5 models are shown. **D-E**. Variable weights from a Random forest correlation model linking (**D**) copy number variation [CNV] and somatic mutations [SNP:ID] or (**E**) copy number variation, gene expression [mRNA] and somatic mutations from the TCGA database to RND3 expression. Percentage of variance in RND3 expression explained: D– 13.97%, E– 21.04%. Plus or minus symbols indicate sign of the Spearman correlation value between RND3 and CNV/mRNA expression.
(TIF)

S1 Text A novel modularization approach to integrating multiple functional genomics datasets. This document describes the background, methodology and performance of the novel multi-level data integration approach used in this study to generate the networks in Fig 6A.
(DOCX)

S1 Table Functional enrichment analysis reveals common terms linked to genes correlated with RND3 expression and/or modulated by RND3 silencing. Significant enrichment indicated by bold text (false discovery rate < 10%).
(TIF)

## References

[1] Maher EA, Furnari FB, Bachoo RM, Rowitch DH, Louis DN, Cavenee WK, et al Malignant glioma: genetics and biology of a grave matter. Genes Dev. 2001;15: 1311–33. 10.1101/gad.891601 11390353

[2] Claus EB, Black PM. Survival rates and patterns of care for patients diagnosed with supratentorial low-grade gliomas: data from the SEER program, 1973–2001. Cancer. 2006;106: 1358–63. 10.1002/cncr.21733 16470608

[3] Furnari FB, Fenton T, Bachoo RM, Mukasa A, Stommel JM, Stegh A, et al Malignant astrocytic glioma: genetics, biology, and paths to treatment. Genes Dev. 2007;21: 2683–710. 10.1101/gad.1596707 17974913

[4] Ohgaki H, Dessen P, Jourde B, Horstmann S, Nishikawa T, Di Patre P-L, et al Genetic pathways to glioblastoma: a population-based study. Cancer Res. 2004;64: 6892–9. 10.1158/0008-5472.CAN-04-1337 15466178

[5] Lee JC, Vivanco I, Beroukhim R, Huang JHY, Feng WL, DeBiasi RM, et al Epidermal growth factor receptor activation in glioblastoma through novel missense mutations in the extracellular domain. PLoS Med. 2006;3: 2264–2273. 10.1371/journal.pmed.0030485 PMC1702556 17177598

[6] Fan QW, Cheng C, Gustafson WC, Charron E, Zipper P, Wong R, et al EGFR Phosphorylates Tumor-Derived EGFRvIII Driving STAT3/5 and Progression in Glioblastoma. Cancer Cell. 2013;24: 438–449. 10.1016/j.ccr.2013.09.004 24135280 PMC3819146

[7] Clarke ID, Dirks PB. A human brain tumor-derived PDGFR-alpha deletion mutant is transforming. Oncogene. 2003;22: 722–733. 10.1038/sj.onc.1206160 12569364

[8] Verhaak RGW, Hoadley KA, Purdom E, Wang V, Qi Y, Wilkerson MD, et al Integrated genomic analysis identifies clinically relevant subtypes of glioblastoma characterized by abnormalities in PDGFRA, IDH1, EGFR, and NF1. Cancer Cell. 2010;17: 98–110. 10.1016/j.ccr.2009.12.020 20129251 PMC2818769

[9] Talasila KM, Soentgerath A, Euskirchen P, Rosland G V, Wang J, Huszthy PC, et al EGFR wild-type amplification and activation promote invasion and development of glioblastoma independent of angiogenesis. Acta Neuropathol. 2013;125: 683–98. 10.1007/s00401-013-1101-1 23429996 PMC3631314

[10] Roberts PJ, Der CJ. Targeting the Raf-MEK-ERK mitogen-activated protein kinase cascade for the treatment of cancer. Oncogene. 2007;26: 3291–3310. 10.1038/sj.onc.1210422 17496923

[11] Holland EC, Celestino J, Dai C, Schaefer L, Sawaya RE, Fuller GN. Combined activation of Ras and Akt in neural progenitors induces glioblastoma formation in mice. Nat Genet. Nature Publishing Group; 2000;25: 55–7. 10.1038/75596 10802656

[12] Salhia B, Tran NL, Chan A, Wolf A, Nakada M, Rutka F, et al The guanine nucleotide exchange factors trio, Ect2, and Vav3 mediate the invasive behavior of glioblastoma. Am J Pathol. 2008;173: 1828–1838. 10.2353/ajpath.2008.080043 19008376 PMC2626393

[13] Katoh H, Hiramoto K, Negishi M. Activation of Rac1 by RhoG regulates cell migration. J Cell Sci. 2006;119: 56–65. 10.1242/jcs.02720 16339170

[14] Kwiatkowska A, Didier S, Fortin S, Chuang Y, White T, Berens ME, et al The small GTPase RhoG mediates glioblastoma cell invasion. Mol Cancer. 2012;11: 65 10.1186/1476-4598-11-65 22966858 PMC3557187

[15] Yan B, Chour HH, Peh BK, Lim C, Salto-Tellez M. RhoA protein expression correlates positively with degree of malignancy in astrocytomas. Neurosci Lett. 2006;407: 124–126. 10.1016/j.neulet.2006.08.032 16978776

[16] Hagedorn M, Javerzat S, Gilges D, Meyre A, de Lafarge B, Eichmann A, et al Accessing key steps of human tumor progression in vivo by using an avian embryo model. Proc Natl Acad Sci U S A. 2005;102: 1643–8. 10.1073/pnas.0408622102 15665100 PMC547849

[17] Santarius T, Shipley J, Brewer D, Stratton MR, Cooper CS. A census of amplified and overexpressed human cancer genes. Nat Rev Cancer. 2010;10: 59–64. 10.1038/nrc2771 20029424

[18] Futreal PA, Coin L, Marshall M, Down T, Hubbard T, Wooster R, et al A census of human cancer genes. Nat Rev Cancer. 2004;4: 177–183. 10.1038/nrc1299 14993899 PMC2665285

[19] Danussi C, Akavia UD, Niola F, Jovic A, Lasorella A, Pe’er D, et al RHPN2 drives mesenchymal transformation in malignant glioma by triggering RhoA activation. Cancer Res. 2013;73: 5140–50. 10.1158/0008-5472.CAN-13-1168-T 23774217 PMC3805507

[20] Salhia B, Rutten F, Nakada M, Beaudry C, Berens M, Kwan A, et al Inhibition of Rho-kinase affects astrocytoma morphology, motility, and invasion through activation of Rac1. Cancer Res. 2005;65: 8792–8800. 10.1158/0008-5472.CAN-05-0160 16204049

[21] Reyes SB, Narayanan AS, Lee HS, Tchaicha JH, Aldape KD, Lang FF, et al αvβ8 integrin interacts with RhoGDI1 to regulate Rac1 and Cdc42 activation and drive glioblastoma cell invasion. Mol Biol Cell. 2013;24: 474–82. 10.1091/mbc.E12-07-0521 23283986 PMC3571870

[22] Fortin SP, Ennis MJ, Schumacher CA, Zylstra-Diegel CR, Williams BO, Ross JTD, et al Cdc42 and the guanine nucleotide exchange factors Ect2 and trio mediate Fn14-induced migration and invasion of glioblastoma cells. Mol Cancer Res. 2012;10: 958–968. 10.1158/1541-7786.MCR-11-0616 22571869 PMC3516844

[23] Riento K, Guasch RM, Garg R, Jin B, Ridley AJ. RhoE binds to ROCK I and inhibits downstream signaling. Mol Cell Biol. 2003;23: 4219–29. Available: http://www.pubmedcentral.nih.gov/articlerender.fcgi?artid=156133&tool=pmcentrez&rendertype=abstract 12773565 10.1128/MCB.23.12.4219-4229.2003 PMC156133

[24] Chardin P. Function and regulation of Rnd proteins. Nat Rev Mol Cell Biol. 2006;7: 54–62. 10.1038/nrm1788 16493413

[25] Lonjedo M, Poch E, Mocholí E, Hernández-Sánchez M, Ivorra C, Franke TF, et al The Rho family member RhoE interacts with Skp2 and is degraded at the proteasome during cell cycle progression. J Biol Chem. 2013;288: 30872–82. 10.1074/jbc.M113.511105 24045951 PMC3829402

[26] J M, B B, D B, B D, P K, M L, et al Regulation of Rnd3 localization and function by protein kinase Calpha-mediated phosphorylation. Portland Press Ltd.; 2009; Available: http://www.biochemj.org/bj/424/bj4240153.htm 10.1042/BJ20082377 PMC2868966 19723022

[27] Zhu Z, Todorova K, Lee KK, Wang J, Kwon E, Kehayov I, et al Small GTPase RhoE/Rnd3 Is a Critical Regulator of Notch1 Signaling. Cancer Res. 2014;74: 2082–93. 10.1158/0008-5472.CAN-12-0452 24525741 PMC4031027

[28] Li J, Deng M, Wei Q, Liu T, Tong X, Ye X. Phosphorylation of MCM3 protein by cyclin E/cyclin-dependent kinase 2 (Cdk2) regulates its function in cell cycle. J Biol Chem. 2011;286: 39776–85. 10.1074/jbc.M111.226464 21965652 PMC3220541

[29] Madhavan S, Zenklusen J-C, Kotliarov Y, Sahni H, Fine HA, Buetow K. Rembrandt: helping personalized medicine become a reality through integrative translational research. Mol Cancer Res. 2009;7: 157–67. 10.1158/1541-7786.MCR-08-0435 19208739 PMC2645472

[30] Heng JI-T, Nguyen L, Castro DS, Zimmer C, Wildner H, Armant O, et al Neurogenin 2 controls cortical neuron migration through regulation of Rnd2. Nature. 2008;455: 114–118. 10.1038/nature07198 18690213

[31] Murphy C, Saffrich R, Olivo-Marin JC, Giner A, Ansorge W, Fotsis T, et al Dual function of rhoD in vesicular movement and cell motility. Eur J Cell Biol. 2001;80: 391–398. 10.1078/0171-9335-00173 11484930

[32] Ellis S, Mellor H. The novel Rho-family GTPase Rif regulates coordinated actin-based membrane rearrangements. Curr Biol. 2000;10: 1387–1390. 10.1016/S0960-9822(00)00777-6 11084341

[33] Gampel A, Parker PJ, Mellor H. Regulation of epidermal growth factor receptor traffic by the small GTPase RhoB. Curr Biol. 1999;9: 955–958. 10.1016/S0960-9822(99)80422-9 10508588

[34] Huang M, Duhadaway JB, Prendergast GC, Laury-Kleintop LD. RhoB regulates PDGFR-beta trafficking and signaling in vascular smooth muscle cells. Arterioscler Thromb Vasc Biol. 2007;27: 2597–2605. 10.1161/ATVBAHA.107.154211 17951322 PMC4384698

[35] Matthys A, Van Craenenbroeck K, Lintermans B, Haegeman G, Vanhoenacker P. RhoBTB3 interacts with the 5-HT7a receptor and inhibits its proteasomal degradation. Cell Signal. 2012;24: 1053–1063. 10.1016/j.cellsig.2011.12.027 22245496

[36] Lu A, Pfeffer SR. Golgi-associated RhoBTB3 targets cyclin E for ubiquitylation and promotes cell cycle progression. J Cell Biol. 2013;203: 233–50. 10.1083/jcb.201305158 24145166 PMC3812982

[37] Baldwin RM, Parolin DAE, Lorimer IAJ. Regulation of glioblastoma cell invasion by PKC iota and RhoB. Oncogene. 2008;27: 3587–95. 10.1038/sj.onc.1211027 18212741

[38] Ma Y, Gong Y, Cheng Z, Loganathan S, Kao C, Sarkaria JN, et al Critical functions of RhoB in support of glioblastoma tumorigenesis. Neuro Oncol. 2014; 10.1093/neuonc/nou228 PMC4483068 25216671

[39] Chan AY, Coniglio SJ, Chuang Y, Michaelson D, Knaus UG, Philips MR, et al Roles of the Rac1 and Rac3 GTPases in human tumor cell invasion. Oncogene. 2005;24: 7821–7829. 10.1038/sj.onc.1208909 16027728

[40] Zhang H, Zhu W, Su X, Wu S, Lin Y, Li J, et al Triptolide inhibits proliferation and invasion of malignant glioma cells. J Neurooncol. 2012;109: 53–62. 10.1007/s11060-012-0885-5 22562416

[41] Khalil BD, Hanna S, Saykali BA, El-Sitt S, Nasrallah A, Marston D, et al The regulation of RhoA at focal adhesions by StarD13 is important for astrocytoma cell motility. Exp Cell Res. 2014;321: 109–22. 10.1016/j.yexcr.2013.11.023 24333506 PMC4297755

[42] Sasayama T, Nishihara M, Kondoh T, Hosoda K, Kohmura E. MicroRNA-10b is overexpressed in malignant glioma and associated with tumor invasive factors, uPAR and RhoC. Int J Cancer. 2009;125: 1407–13. 10.1002/ijc.24522 19536818

[43] Goldberg L, Kloog Y. A Ras inhibitor tilts the balance between Rac and Rho and blocks phosphatidylinositol 3-kinase-dependent glioblastoma cell migration. Cancer Res. 2006;66: 11709–17. 10.1158/0008-5472.CAN-06-1878 17178866

[44] Yoon C-H, Hyun K-H, Kim R-K, Lee H, Lim E-J, Chung H-Y, et al The small GTPase Rac1 is involved in the maintenance of stemness and malignancies in glioma stem-like cells. FEBS Lett. 2011;585: 2331–8. 10.1016/j.febslet.2011.05.070 21704033

[45] Zhou X, Qian J, Hua L, Shi Q, Liu Z, Xu Y, et al Geranylgeranyltransferase I promotes human glioma cell growth through Rac1 membrane association and activation. J Mol Neurosci. 2013;49: 130–9. 10.1007/s12031-012-9905-3 23073905

[46] Poch E, Miñambres R, Mocholí E, Ivorra C, Pérez-Aragó A, Guerri C, et al RhoE interferes with Rb inactivation and regulates the proliferation and survival of the U87 human glioblastoma cell line. Exp Cell Res. 2007;313: 719–31. 10.1016/j.yexcr.2006.11.006 17182035

[47] Ongusaha PP, Kim H-G, Boswell SA, Ridley AJ, Der CJ, Dotto GP, et al RhoE is a pro-survival p53 target gene that inhibits ROCK I-mediated apoptosis in response to genotoxic stress. Curr Biol. 2006;16: 2466–72. 10.1016/j.cub.2006.10.056 17174923 PMC2779528

[48] Bektic J, Pfeil K, Berger AP, Ramoner R, Pelzer A, Schäfer G, et al Small G-protein RhoE is underexpressed in prostate cancer and induces cell cycle arrest and apoptosis. Prostate. 2005;64: 332–40. 10.1002/pros.20243 15754346

[49] Sun L, Hui A-M, Su Q, Vortmeyer A, Kotliarov Y, Pastorino S, et al Neuronal and glioma-derived stem cell factor induces angiogenesis within the brain Cancer Cell. Neuro-Oncology Branch, National Cancer Institute/National Institute of Neurological Disorders and Stroke, National Institutes of Health, Bethesda, Maryland 20892, USA.; 2006;9: 287–300. 10.1016/j.ccr.2006.03.003 16616334

[50] Irizarry RA, Hobbs B, Collin F, Beazer-Barclay YD, Antonellis KJ, Scherf U, et al Exploration, normalization, and summaries of high density oligonucleotide array probe level data. Biostat Oxford Engl. 2003;4: 249–264. Available: http://www.ncbi.nlm.nih.gov/pubmed/12925520 10.1093/biostatistics/4.2.249 12925520

[51] Benjamini Y, Hochberg Y. Controlling the false discovery rate: a practical and powerful approach to multiple testing. J R Stat Soc Ser B Methodol. 1995;57: 289–300. 10.2307/2346101

[52] Subramanian A, Tamayo P, Mootha VK, Mukherjee S, Ebert BL, Gillette MA, et al Gene set enrichment analysis: a knowledge-based approach for interpreting genome-wide expression profiles. Proc Natl Acad Sci U S A. National Academy of Sciences; 2005;102: 15545–15550. 10.1073/pnas.0506580102 PMC1239896 16199517

[53] Tarcea V, Weymouth T, Ade A. Michigan molecular interactions r2: from interacting proteins to pathways. Nucleic acids …. 2009;37 10.1093/nar/gkn722 PMC2686565 18978014

[54] Stark C, Breitkreutz B-J, Reguly T, Boucher L, Breitkreutz A, Tyers M. BioGRID: a general repository for interaction datasets. Nucleic Acids Res. 2006;34: D535–D539. 10.1093/nar/gkj109 16381927 PMC1347471

[55] Peri S, Navarro JD, Amanchy R, Kristiansen TZ, Jonnalagadda CK, Surendranath V, et al Development of human protein reference database as an initial platform for approaching systems biology in humans. Genome Res. 2003;13: 2363–2371. 10.1101/gr.1680803 14525934 PMC403728

[56] Chin C-S, Samanta MP. Global snapshot of a protein interaction network-a percolation based approach. Bioinformatics. 2003;19: 2413–2419. 10.1093/bioinformatics/btg339 14668225

[57] Su G, Kuchinsky A, Morris JH, States DJ, Meng F. GLay: community structure analysis of biological networks. Bioinformatics. 2010;26: 3135–3137. 10.1093/bioinformatics/btq596 21123224 PMC2995124

[58] Margolin A, Wang K, Lim W, Kustagi M, Nemenman I, Califano A. Reverse engineering cellular networks Nat Protoc. Department of Biomedical Informatics, Columbia University, New York, New York 10032, USA.: Nature Publishing Group; 2006;1: 662–671. 10.1038/nprot.2006.106 17406294

[59] Van Der Laan M J. A new algorithm for hybrid hierarchical clustering with visualization and the bootstrap. J Stat Plan Inference. 2003;117: 275–303. 10.1016/S0378-3758(02)00388-9

[60] Ryan KR, Lock FE, Heath JK, Hotchin NA. Plakoglobin-dependent regulation of keratinocyte apoptosis by Rnd3. J Cell Sci. 2012;125: 3202–9. 10.1242/jcs.101931 22454524

[61] Lock FE, Hotchin NA. Distinct roles for ROCK1 and ROCK2 in the regulation of keratinocyte differentiation. PLoS One. 2009;4: e8190 10.1371/journal.pone.0008190 19997641 PMC2780731

[62] Aryee MJ, Gutiérrez-Pabello JA, Kramnik I, Maiti T, Quackenbush J. An improved empirical bayes approach to estimating differential gene expression in microarray time-course data: BETR (Bayesian Estimation of Temporal Regulation). BMC Bioinformatics. 2009;10: 409 10.1186/1471-2105-10-409 20003283 PMC2801687

[63] Tusher VG, Tibshirani R, Chu G. Significance analysis of microarrays applied to the ionizing radiation response. Proc Natl Acad Sci U S A. 2001;98: 5116–5121. 10.1073/pnas.091062498 11309499 PMC33173

[64] Dittrich MT, Klau GW, Rosenwald A, Dandekar T, Muller T. Identifying functional modules in protein-protein interaction networks: An integrated exact approach. Bioinformatics. 2008;24 10.1093/bioinformatics/btn161 PMC2718639 18586718

