# Supplementary figures and images for: Inference of Low and High-Grade Glioma Gene Regulatory Networks Delineates the Role of Rnd3 in Establishing Multiple Hallmarks of Cancer
Source: PLoS Genet. 2015 Jul 1;11(7):e1005325. doi: 10.1371/journal.pgen.1005325 (PMC4488580; doi:10.1371/journal.pgen.1005325)

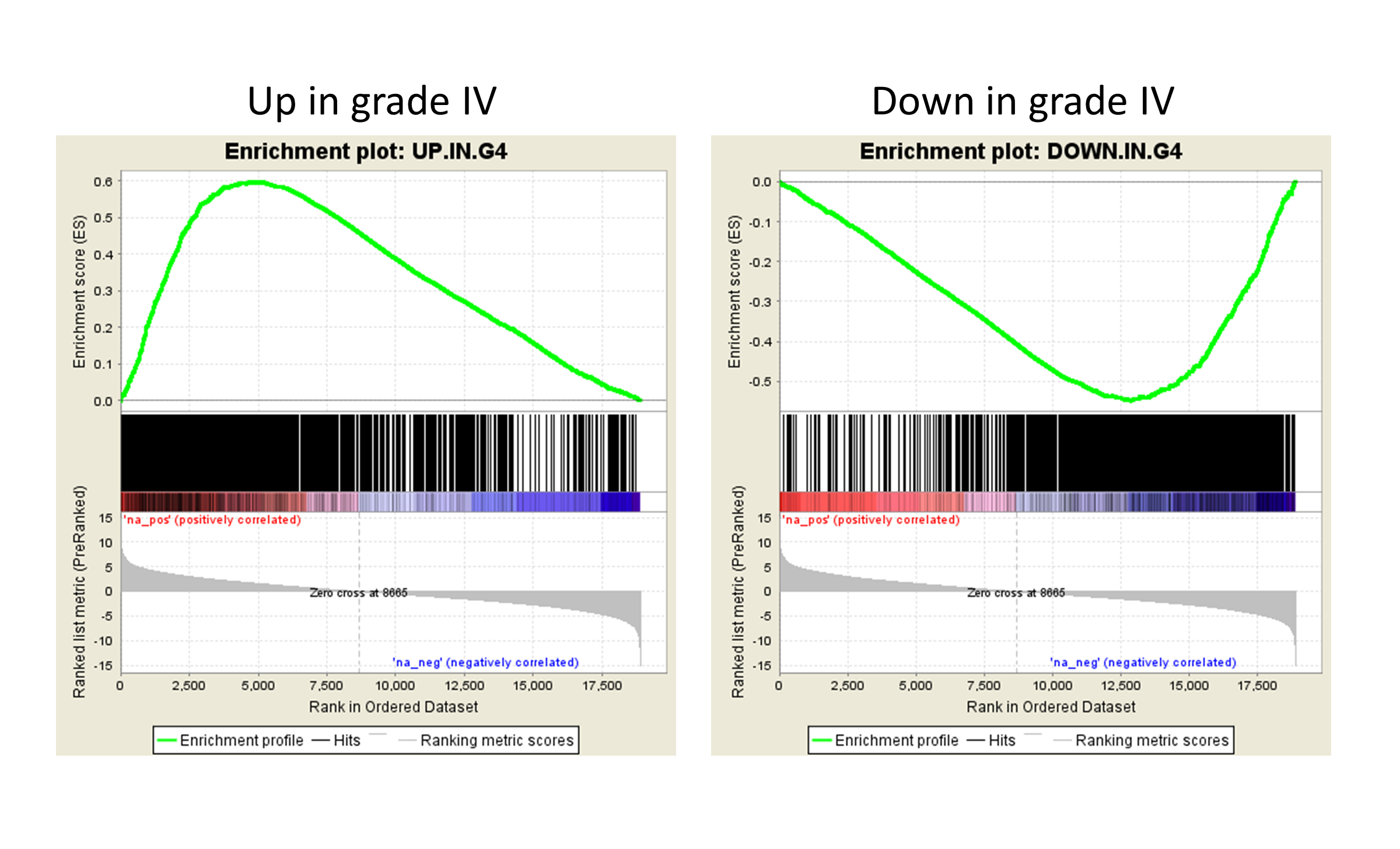

Supplement: S1 Fig — Enrichment score (ES) plots generated by comparing differentially expressed genes between grade II and grade IV glioma in GSE4290 and GSE52009 datasets using the Gene Set Enrichment Analysis software. False discovery rate <0.001% for both comparisons. (TIF) [file pgen.1005325.s006.tif]

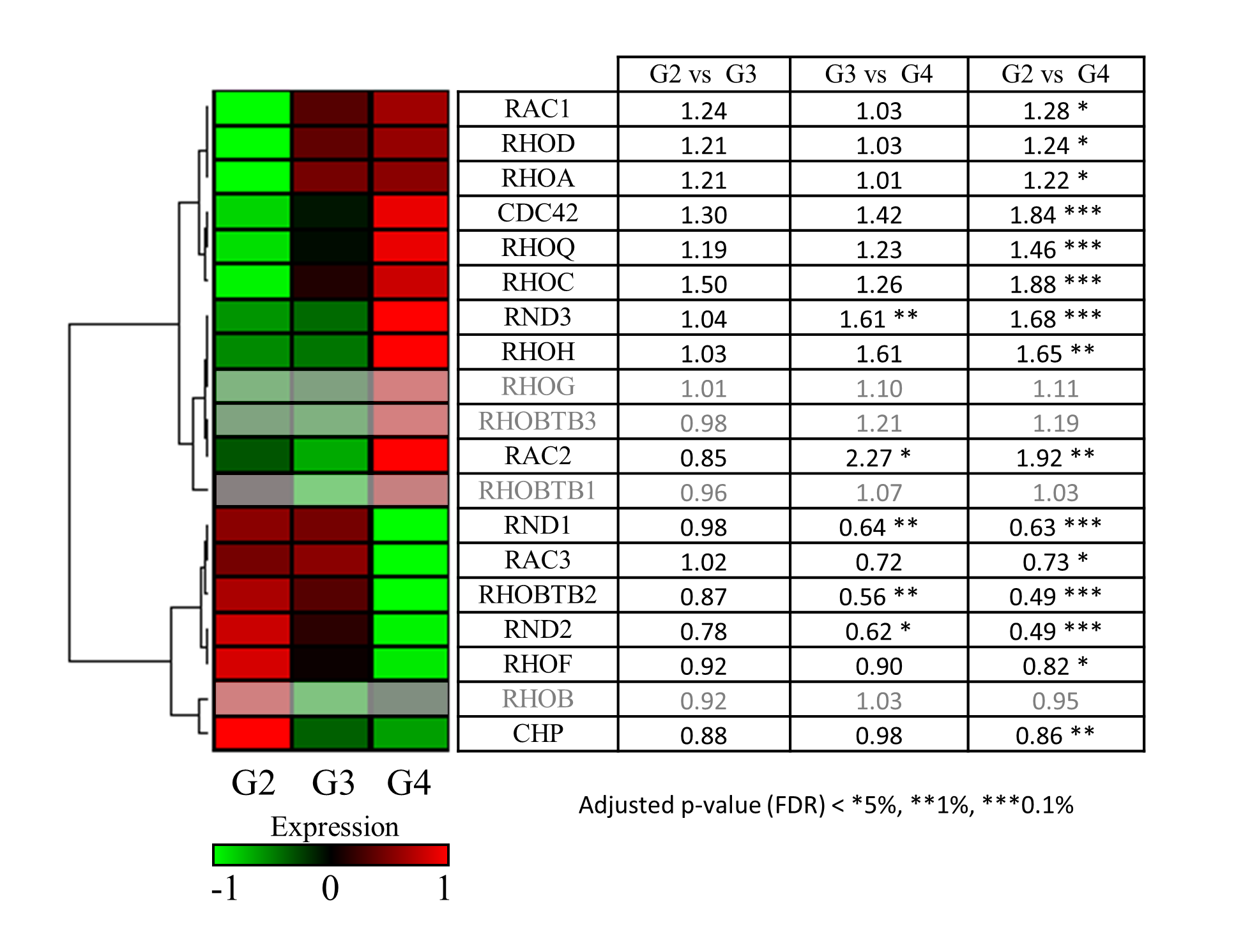

Supplement: S2 Fig — The expression of Rho GTPases in the Sun et al dataset is represented as a colour heatmap. Gene expression is standardised by row for visualisation. Mean linear fold change values are indicated for each glioma grade comparison. Statistical significance is determined by t-test followed by Benjamini-Hochberg FDR correction. (TIF) [file pgen.1005325.s007.tif]

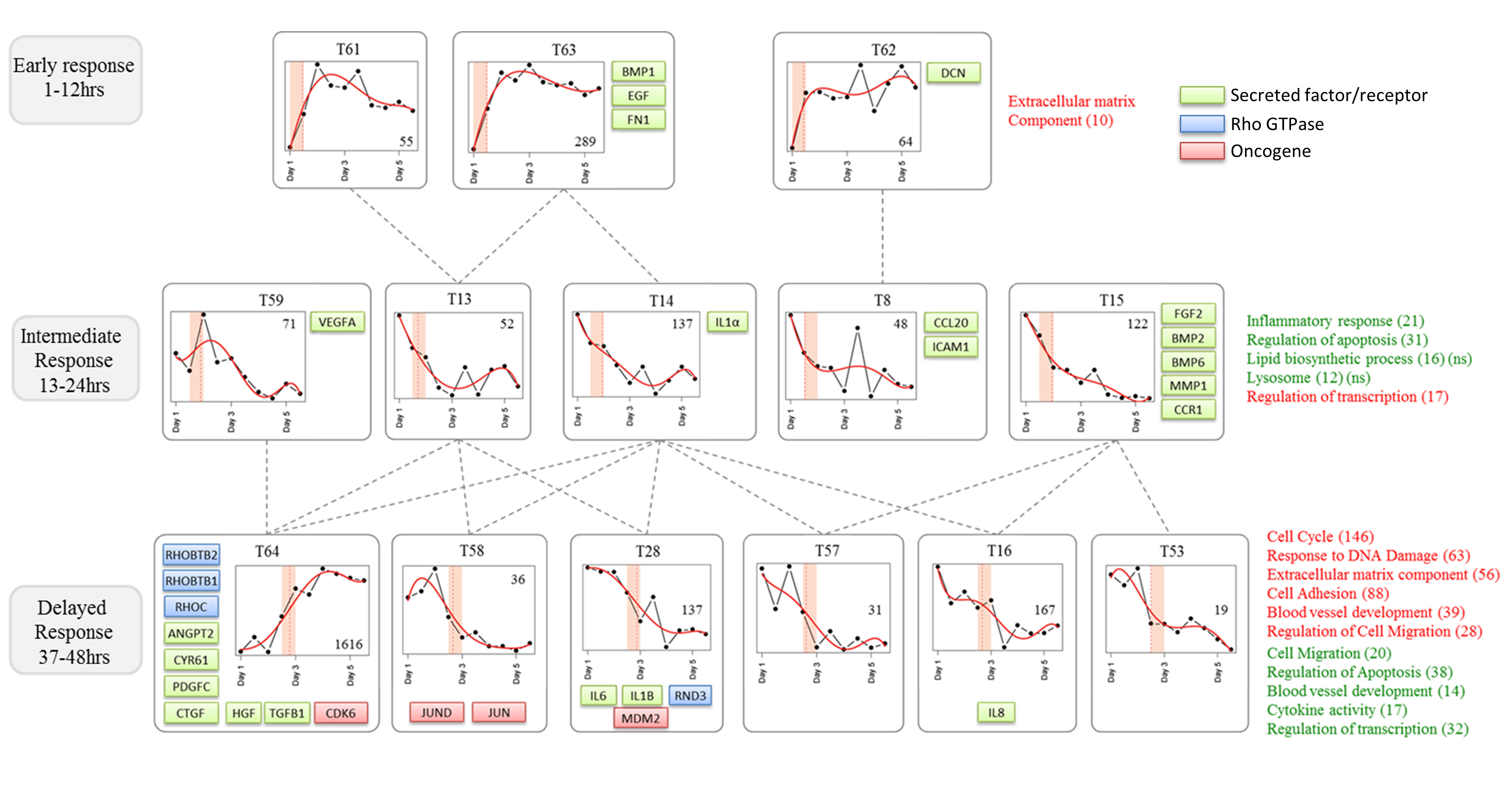

Supplement: S3 Fig — 14 distinct gene clusters are categorised as early (1–12hrs; top row), intermediate (13–24hrs; middle row) or delayed responders (37–48hrs; bottom row) according to the time point at which the gene expression is altered by 50% of the dynamic range of the cluster. The time window (pink bar) and exact position in the interpolated time series (dotted line within pink bar) at which the cluster has altered by 50% of the clusters dynamic range is shown in respect to the medoid expression profile of each cluster (black line) and the interpolated expression profile (red line). Significant time-delay correlations between expression profiles are shown as dotted lines between clusters. Genes within each group of clusters (early, intermediate, and delayed) were determined to be up or down-regulated according to the expression trend of the cluster. A representative selection of Gene Ontology terms enriched within up (red) and down-regulated (green) genes at each level are coloured accordingly (false discovery rate < 10%). ns: non-significant. Individual genes within each cluster are coloured according to function: Rho GTPase (blue), secreted factor (green) and known oncogene (red). (TIF) [file pgen.1005325.s008.tif]

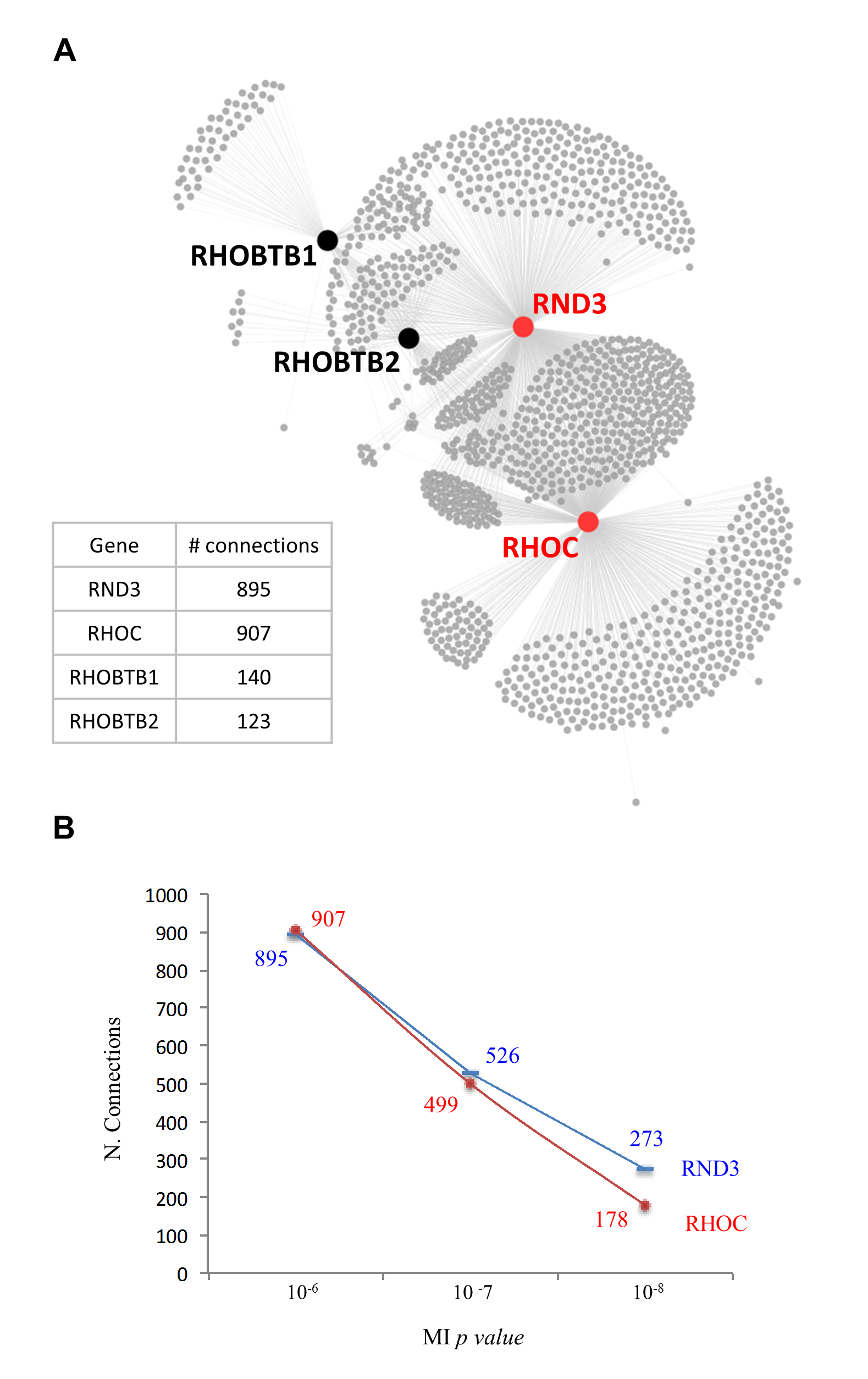

Supplement: S4 Fig — A. The gene neighbourhood of Rho GTPases RHOBTB1, RHOBTB2, RND3 and RHOC in the CAM network (mutual information p value < 10−6). B. The size of the gene neighbourhood of RND3 and RHOC at a range of high statistical thresholds. (TIF) [file pgen.1005325.s009.tif]

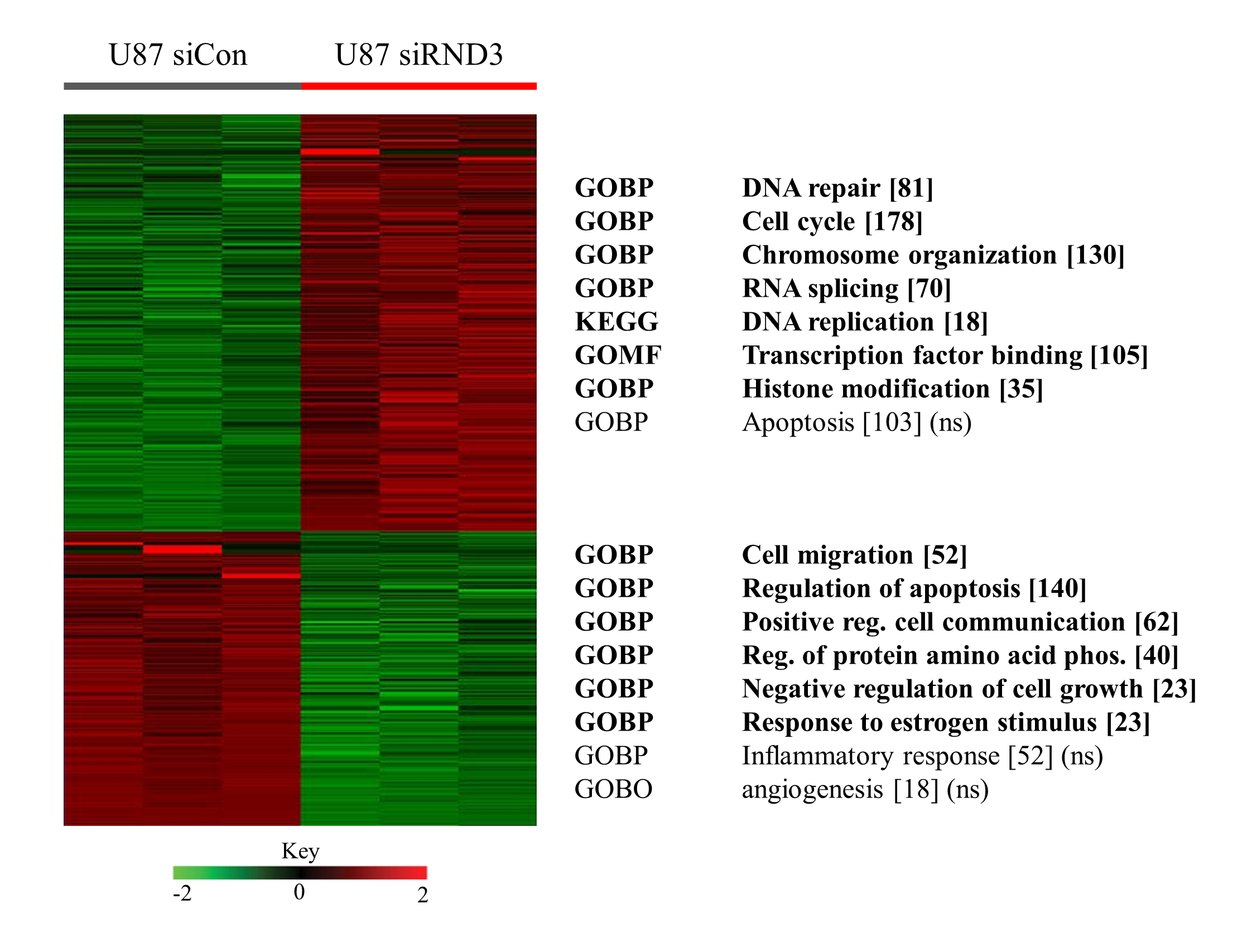

Supplement: S5 Fig — Heatmap representing expression profiles of differentially expressed genes between U87 siControl (n = 3) and U87 siRND3 (n = 3) cells. The number of genes matched to Gene Ontology terms within up and down-regulated genes are shown. Terms in bold are significantly enriched (false discovery rate < 10%). ns = non-significant. (TIF) [file pgen.1005325.s010.tif]

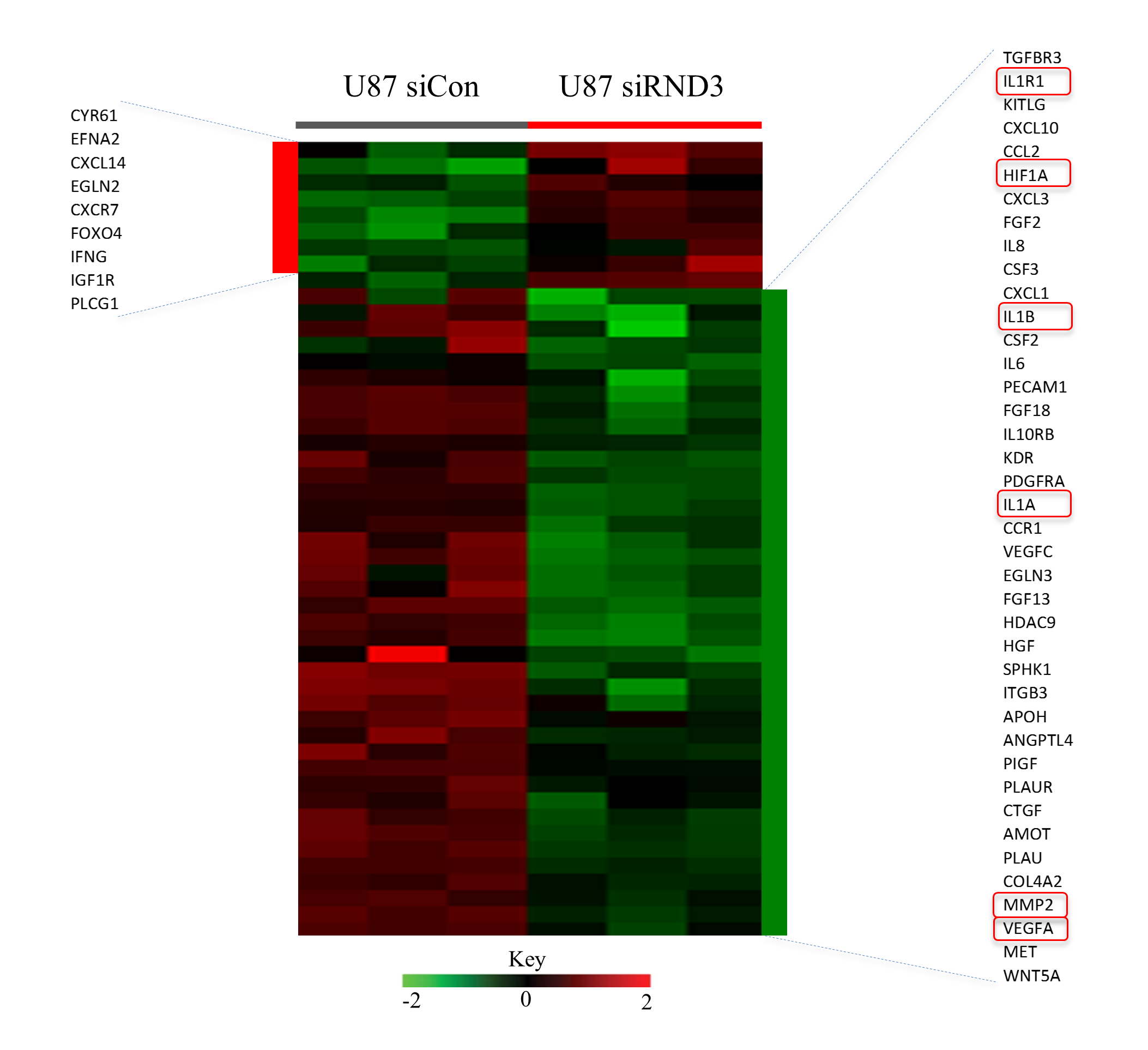

Supplement: S6 Fig — Heatmap representing expression profiles of a selection of differentially expressed genes related to extracellular processes. (TIF) [file pgen.1005325.s011.tif]

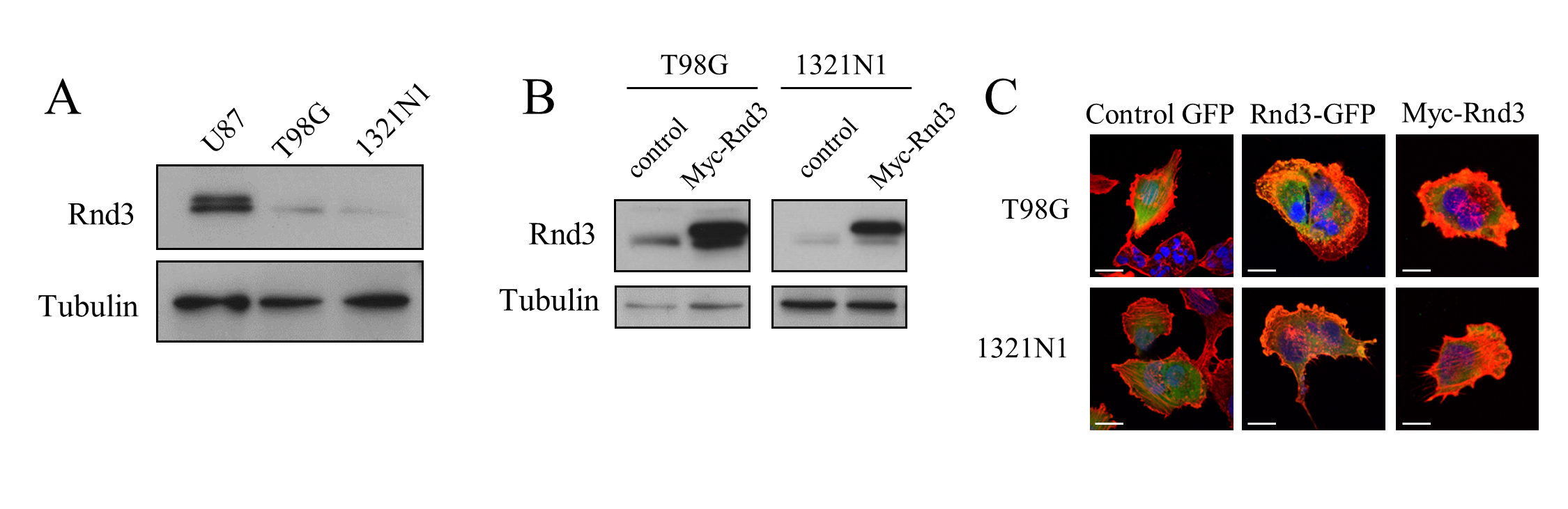

Supplement: S7 Fig — A. Western blot analyses with RND3 and tubulin antibodies on U87, T98G and 1321N1 cells. B. Western blot analyses with RND3 and tubulin antibodies on myc-RND3 expressing T98G and 1321N1 cells. C. Ectopic expression of RND3-GFP or myc-RND3 in T98G and 1321N1 cells. RND3-GFP or myc-RND3 are represented in green and F-actin/DAPI stainings in red and blue respectively. Scale bars, 10 μm. (TIF) [file pgen.1005325.s012.tif]

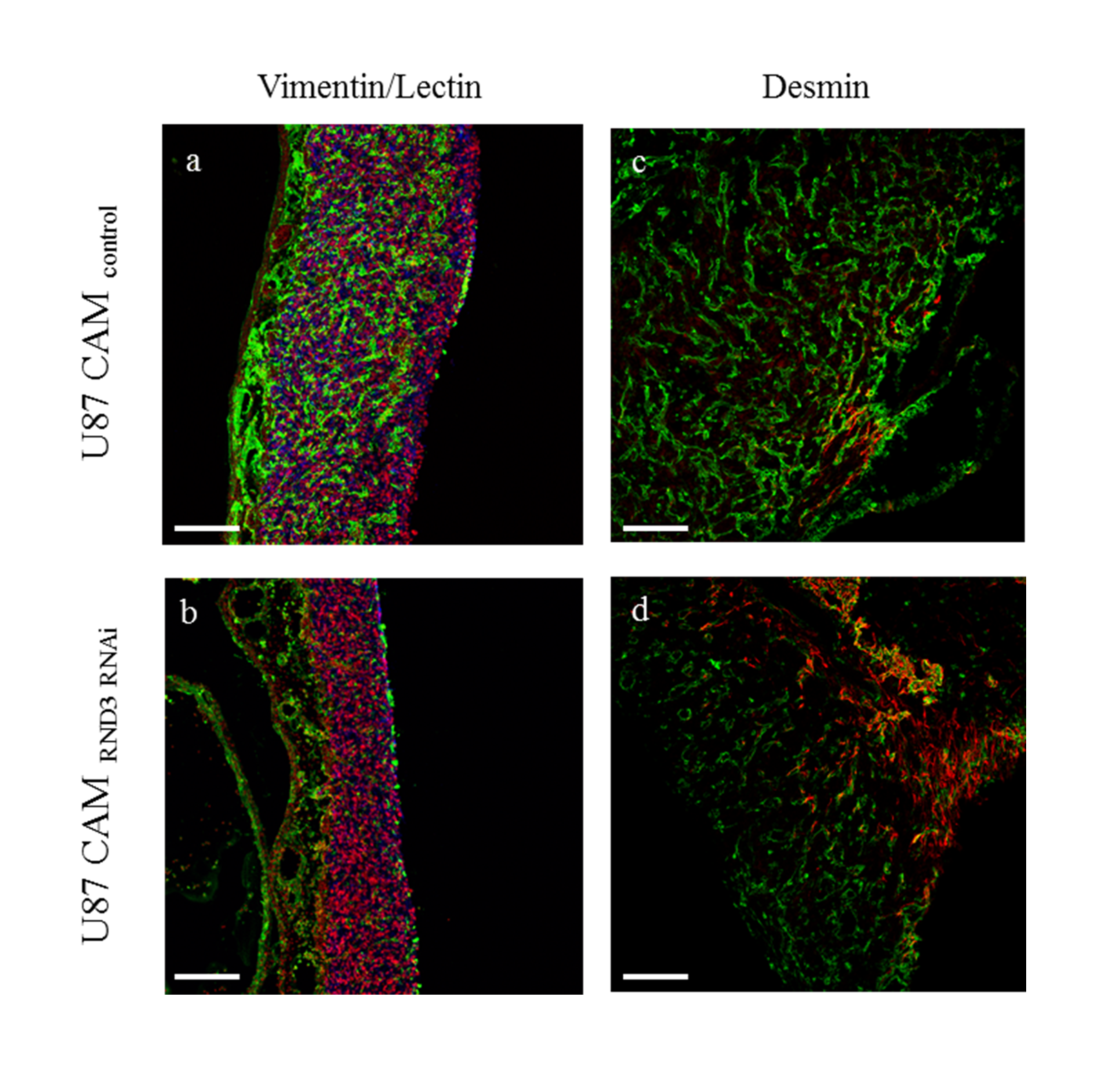

Supplement: S8 Fig — Biomicroscopic pictures from tumors grown on the CAM. A-D. Representative immunohistochemistry staining of vimentin (A, B) or desmin (C, D). Tumors derived from U87 siControl (A, C) or U87 siRND3 (B, D) cells. Magnifications: (A, B) x10; scale bar 200μm, (C, D) x20; scale bar 100μm. (TIF) [file pgen.1005325.s013.tif]

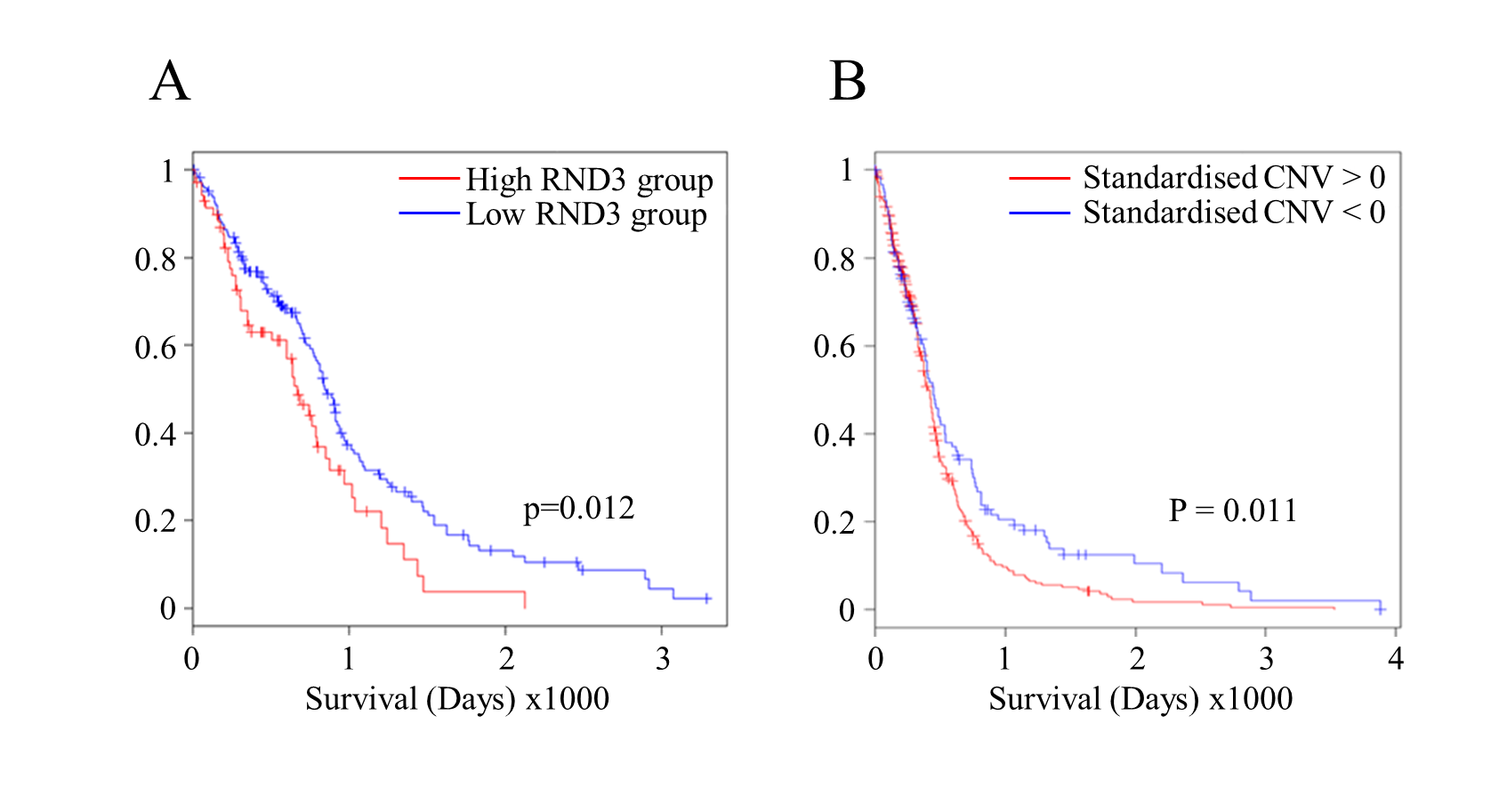

Supplement: S9 Fig — Kaplan-Meier survival curves of glioma patients partitioned by lower or higher RND3 expression and CNV in TCGA datasets. (A) The higher RND3 expression (n = 70) group has significantly lower survival rates than the low RND3 expression group (n = 165), p = 0.012, HR = 1.1–2.2. (B) The higher RND3 CNV (n = 317) group has significantly lower survival than the low RND3 CNV group (n = 119), p = 0.011, HR = 1.1–1.7. (TIF) [file pgen.1005325.s014.tif]

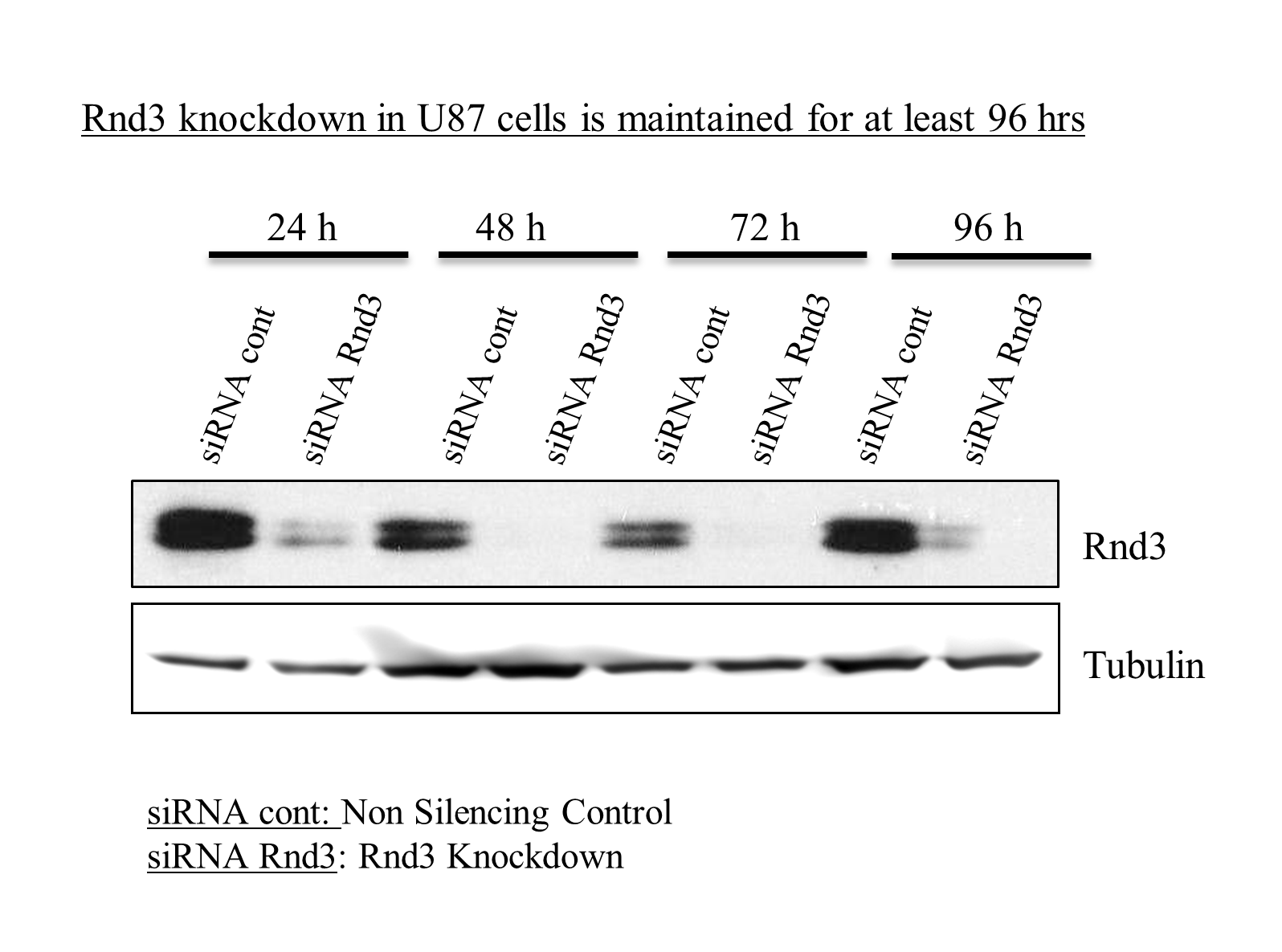

Supplement: S10 Fig — RND3 expression determined by western blot in U87 cells 24, 48, 72 and 96 hours after transfection with RND3 siRNA. (TIF) [file pgen.1005325.s015.tif]

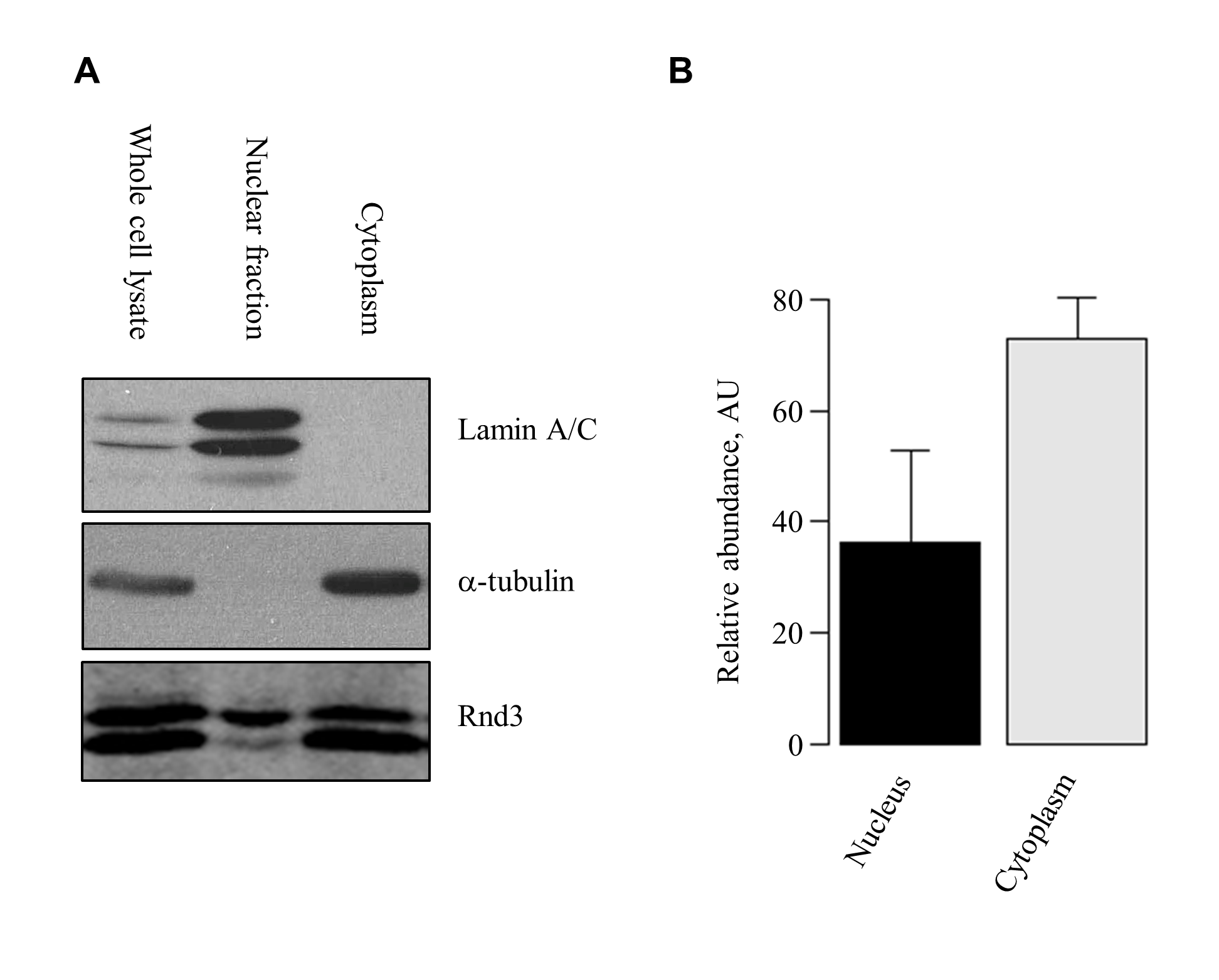

Supplement: S11 Fig — A. Detection of RND3 in whole cell lysates, nuclear fraction and cytoplasmic fraction of U87 cells determined by western blot. Lamin A/C and α-tubulin are included as exclusively nuclear/cytoplasmic controls respectively. B. Quantification of the western blot analysis by densitometry. (TIF) [file pgen.1005325.s016.tif]

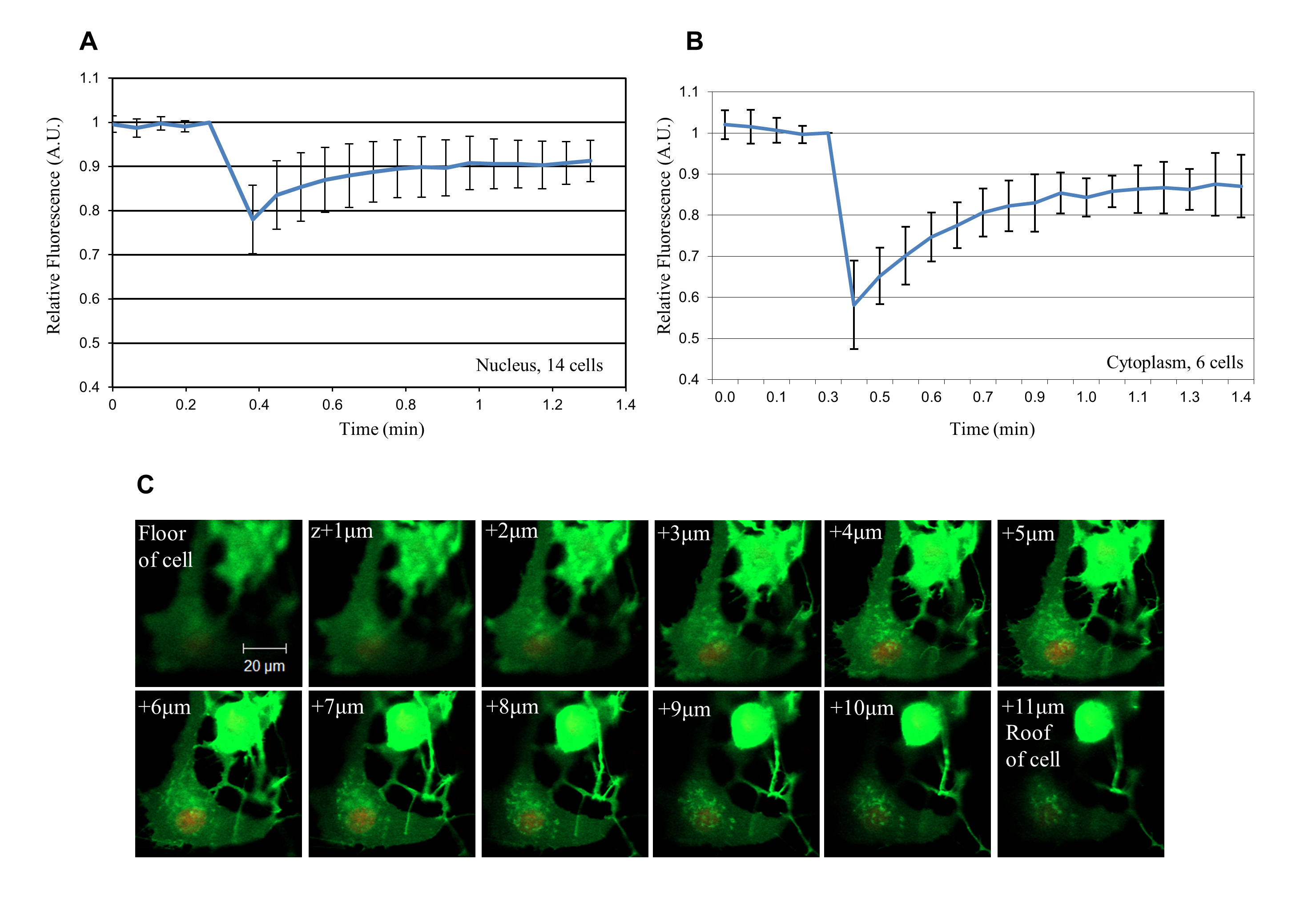

Supplement: S12 Fig — A-B.Fluorescence recovery after photo-bleaching (FRAP) time-course for U87 cells expressing RND3-GFP. Cells were bleached after indicated time point using 20 iterations of 488nm laser set to 100%. Same-sized regions were bleached in the (A) entire nucleus (n = 14 cells) and (B) fraction of the cytoplasm (n = 6 cells). Fluorescence recovery was monitored and data plotted normalised to pre-bleach fluorescence intensity ± standard deviation. C. Image series showing representative U87 cell expressing H2B-mcherry (red) and RND3-GFP (green), imaged in sequential 1um depth ‘slices’ through the cell. (TIF) [file pgen.1005325.s017.tif]

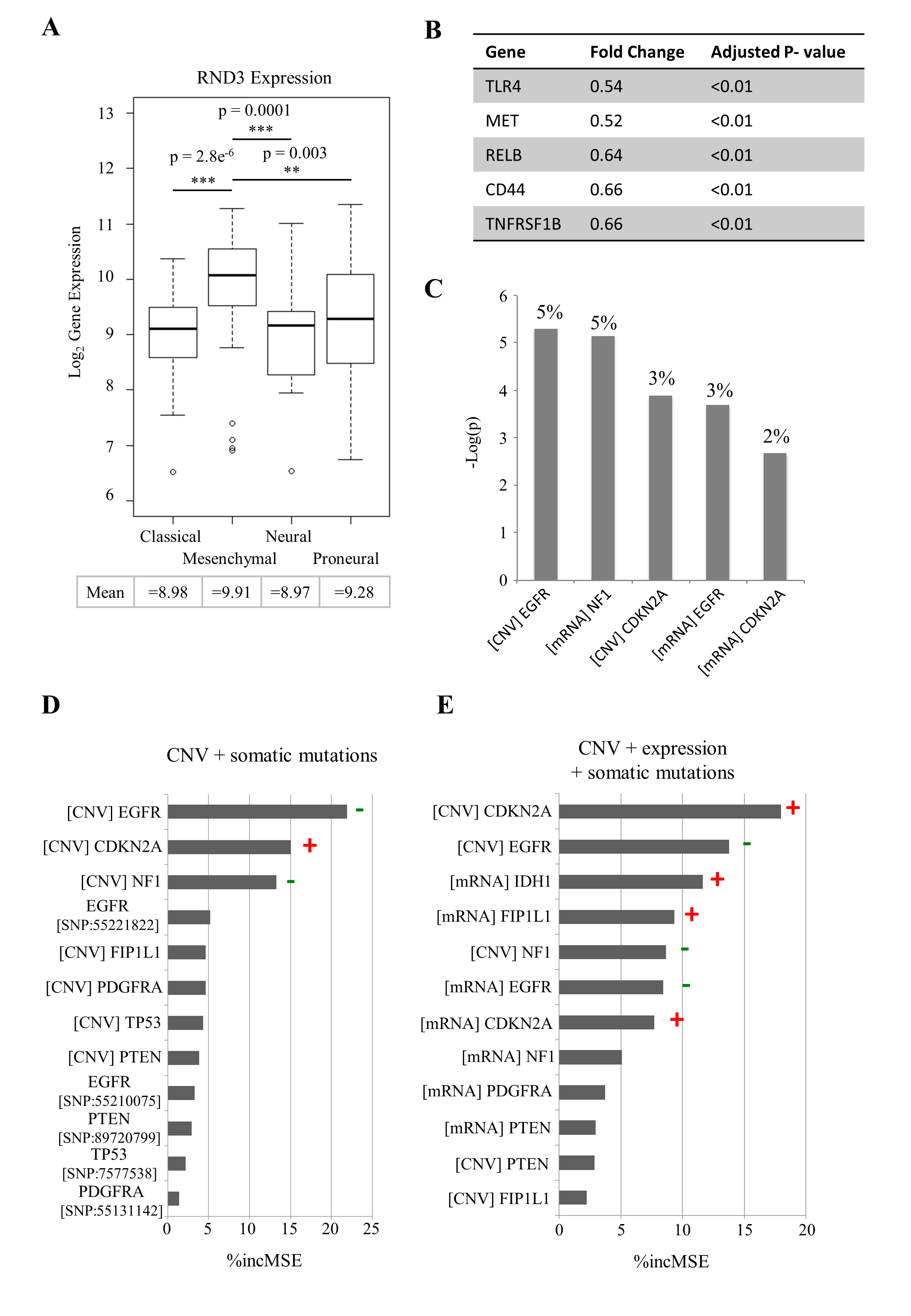

Supplement: S13 Fig — A. RND3 gene expression in patients with glioblastoma subtypes defined by Verhaak et al, 2010. Data consists of glioblastoma patients from the Cancer Genome Atlas database. B. Change in expression of genes characteristic of the mesenchymal subtype in U87 cells after RND3 silencing. C. Significance and percentage of variance in RND3 expression explained by univariate regression analysis using CNV or mRNA levels of frequently mutated genes in GBM. The top 5 models are shown. D-E. Variable weights from a Random forest correlation model linking (D) copy number variation [CNV] and somatic mutations [SNP:ID] or (E) copy number variation, gene expression [mRNA] and somatic mutations from the TCGA database to RND3 expression. Percentage of variance in RND3 expression explained: D– 13.97%, E– 21.04%. Plus or minus symbols indicate sign of the Spearman correlation value between RND3 and CNV/mRNA expression. (TIF) [file pgen.1005325.s018.tif]

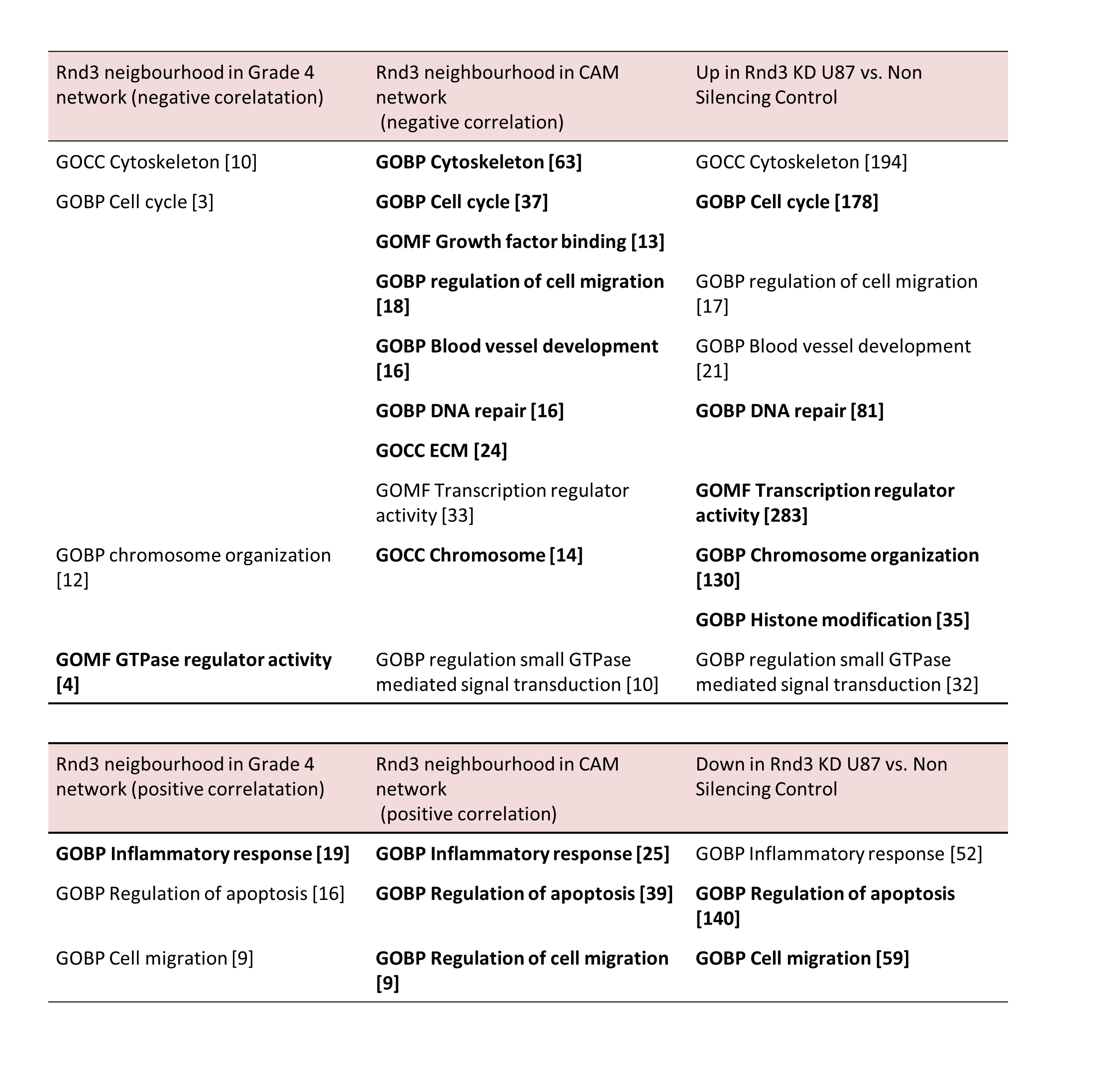

Supplement: S1 Table — Significant enrichment indicated by bold text (false discovery rate < 10%). (TIF) [file pgen.1005325.s020.tif]
