# Supplementary material for: Inference of Low and High-Grade Glioma Gene Regulatory Networks Delineates the Role of Rnd3 in Establishing Multiple Hallmarks of Cancer
Source: PLoS Genet. 2015 Jul 1;11(7):e1005325. doi: 10.1371/journal.pgen.1005325 (PMC4488580; doi:10.1371/journal.pgen.1005325)
Supplement: S1 Text — This document describes the background, methodology and performance of the novel multi-level data integration approach used in this study to generate the networks in Fig 6A. (DOCX) [file pgen.1005325.s019.docx]

# **Text S1: A novel Modularization Approach to Integrating Multiple Functional Genomics Datasets**

## Background

Approaches for identification of sub-networks integrating physical interactions derived from the literature and mRNA expression data has been developed by several groups. These approaches are either aiming at identify sub-networks whose genes are differentially regulated between various experimental conditions [65], [64] or are highly co-regulated [66] [67]. Ideker *et al*. [65] proposed a heuristic approach aiming to identify modules in protein-protein interaction networks that were enriched with differentially expressed genes. In their paper, Ideker *et al.* demonstrated the usefulness of this approach integrating yeast protein interaction with gene expression data measured from 20 perturbations to the yeast galactose utilization pathway. Scott *et al.* addressed the same challenge using a graph theory approach based on Steiner trees [68]. In their method, they applied an approximation algorithm developed by Klein *et al.* [69]. Dittrich *et al*. has also tackled this problem using Steiner Trees, but they have used an algorithm developed by Lubic *et al.* [70], which can solve Steiner Tree problems to optimality. This methodology had excellent performances on the simulated data and out-performed the alternative methodology which used a heuristic search. Other approaches have been developed to identify functional modules, whose members are highly co-regulated [66] [67]. In these methodologies, edges based scoring functions are introduced in order identify modules with high scores.

However, so far a methodology integrating node and edge properties is missing. If available, such procedure would allow the identification of network modules integrating three different levels of information. For example, protein-protein interaction modules that are enriched with genes differentially expressed between two or more experimental groups and also co-regulated across a large numbers of experimental perturbations (e.g. different genetic backgrounds or across a patient population). Here, a new method addressing this challenge is presented, which is derived from the procedure developed by Dittrich *et al.* [64]. The novel methodology has a high sensitivity and specificity on simulated data and its application to the RND3 biological dataset yields relevant hypothesis.

## Methodology

### The Modularization Approach

The proposed methodology aims to identify sub-networks of a larger molecular interaction network, whose elements share several functional properties. Various properties could be used to construct and score the initial network depending on the question to be addressed. In this case, these properties include the integration of three generalised levels of information in a network context. In the presented case study, the p-values derived from the mass spectrometry data representing the targets of Rnd3 protein are used for scoring the nodes and the correlation between the genes during the timecourse of tumour development in the CAM model are used for scoring the edges.

This problem has recently been proven to be equivalent to a Prize-Collecting Steiner Tree Problem (PCST) [68] [64]. Briefly, in a PCST the nodes represents the profits and the edges represent the costs. Networks are scored by computing the sum of the profits subtracted by the sum of the cost of its members. The goal is to identify sub-networks with the highest score. Therefore, the node scoring function should transform low-p-values into higher node profits. Similarly, since highly correlated genes have higher correlation coefficients the edge scoring function should express these gene-to-gene connections with a lower edge cost.

### Constructing interaction networks

The first step is the construction of interaction networks and the selection of the database to use is crucial. A number of databases have been developed in the past few years. Some of them are publicly available (for a review [71]) and others are accessible in the context of commercial software packages [72]. In this application, we have used the BioNet R package in order to access the HPRD database [55] [73] and extract an interaction network consisting of 36504 literature-curated interactions between 9392 different proteins. HPRD is the most comprehensive human protein-protein interaction database being developed to date and contains the large majority of interactions (75%) available in other similar databases such as BIND, DIP and BIOGRID [71].

### Scoring the network

Once the interaction network is constructed, the nodes and the edges in the networks are scored as described below.

**Node Scores**

Each node is scored as a function of a *p-value* derived from a statistical test. This can be the output of any statistical procedure comparing two or more experimental groups. It may also represent a measure of confidence that assign a particular property to a node such as a target gene. The score for each node is defined as:

$score_{g}=-\log(p)$, (1)

where$g$is the score of the gene $g$and $p$is the associate p-value of the gene $g$derived from the differential expression analysis or confidence value. This basic score for each gene allows us to obtain larger scores for smaller p-values.

**Edge Scores**

Each network edge is scored as a function of a correlation coefficient. This can be either a standard correlation coefficient or mutual information. For smaller datasets (*less than* 40 samples) a descriptive measure of dependency such as the Spearman correlation coefficient may be more appropriate whereas MI can be more effective with larger sample size [74].

The edge score $score_{e}$ is defined as:

$score_{e}=-\log(I(g_{i},g_{j}))*w$, (2)

where, *I* is the correlation coefficient (or MI) between genes $g_{i}$ and $g_{j}$, and w is a weight. The final scoring function to maximize is dependent on the edge (2) and node score (1), therefore the contribution of the cost (i.e., edge score) can be increased or decreased using the weight parameter *w* in (2). The size of the identified sub-network depends on this parameter.

In the study case data, the Spearman correlation between genes in the tumour data has been computed in R.

### Scoring sub-networks

The aim of the procedure is to identify the highest sub-networks that are scored by computing the sum of the profits subtracted by the sum of the cost of its members. For a given sub-network A with a set of nodes N and a set of edges E, the module score *score_A_* is then defined as follows:

$score_{A}=\sum_{g\in N} score_{g}-\sum_{e\in E} score_{e}$ **(3)**

The scores of a node $score_{g}$ and the score of an edge $score_{e}$ are computed by applying equations (1) and (2) respectively.

### The algorithm to identify sub-network with highest score in the interaction network

In order to identify the sub-network with highest score in the interaction network, the algorithm developed and implemented in [70] was used. This algorithm has been shown to solve the Price Collecting Steiner Tree (PCST) problems to optimality. For this, they have first transformed the mathematical problem of identifying the sub-network with the maximum score into an equivalent problem called a Steiner arborescence problem. They have shown that a feasible arborescence with minimum total edge corresponds to an optimal PCST. The proofs can be found in [70]. Here, an example is given demonstrating the transformation of the tree in **Figure 1**. To identify the minimum Steiner arborescence tree, they have used integer linear formulation and a branch and cut framework using the software dhea [70]. For this, several types of constraints (connectivity, asymmetry and flow balance) are introduced and violated constraints are identified iteratively until all constraints are satisfied.

This software is implemented using IBM ILOG CPLEX Optimizer, which is a high-performance mathematical programming solver for integer linear programming*.* For academic users, CPLEX is available with no-charge via IBM’s Academic Initiative program.

### Correcting the network score for random effects

It is of crucial importance to test the likelihood of the module of being occurred by chance. On this account, the z-score of the sub-network separately for edges and nodes were computed as follows:

For a given sub-network A of size K =k, an edge z-score is then defined as:

$z\_score\_edges_{A}=\frac{\sum_{e\in A} score_{e}-\text{µ}_{\text{k}}}{\sigma_{k}}$ (4)

where $\text{µ}_{\text{k}}$ and $\sigma_{k}$ are respectively the mean and standard deviation of network edge scores (as defined in (2)) computed for 1000 random sub-networks of size K.

Similarly, for a given sub-network A of size K =k, the node z-score is defined as:

$$z\_score\_nodes_{A}=\frac{\sum_{g\in A} score_{g}-\text{µ}_{\text{k}}}{\sigma_{k}}$$

(5)

where $\text{µ}_{\text{k}}$ and $\sigma_{k}$ are respectively the mean and standard deviation of network node scores (as defined in (1)) computed for 1000 random sub-networks of size *k*. In this application, these were estimated from the module score (calculated using equation (4) and (5)) distribution derived from resampling 1000 random sub-networks of size K.

### Searching for independent multiple sub-networks

The algorithm used ensures identification of the sub-network with the highest score with the given profits and costs [70]. The contribution of the cost to the scoring function can be scaled using the parameter *w* in equation (2) in order to obtain sub-networks with various sizes. It is then possible to compare these networks by their z-scores derived from (4) and (5) and choose the network using the value and the highest z-score divided by the square root of the edge or node size. The chosen sub-network is removed from the interaction network before the search procedure is restarted to identify other independent sub-networks iteratively. These iterations are repeated until there are no networks with acceptable p-values calculated using (3) and (4). The original algorithm can also provide the suboptimal solutions using hamming distance. However, the iterative procedure allows identification of completely independent networks that are relatively small and easy to interpret.


### Method validation on a simulated search space

We have created two simulated search space based on a dataset containing 44000 probes and 54 samples, and its integrated network comprising 4540 genes and 14903 interactions. In one simulated search space, three modules of size 22 nodes were selected randomly and in the second search space three networks of size 55 nodes were selected randomly. Genes belonging to these *a priori* defined modules are set signal p-values uniformly distributed between 0 and 0.10 and background noise p-values uniformly distributed between 0 and 1.

The degree of co-expression between different genes is simulated such that genes are only associated by high MI values if they are part of the same *a priori* defined modules. However, different intervals have been chosen. Modules have relatively low, high and very high pairwise MI values (**Figure 2**).

### Performance of the approach on the simulated data

The identified modules have a recall of 0.8, 0.9 and a precision of 1, 1 for the *a priori* network defined with the highest significance of size 22 (**Figure 3A**) and 55 nodes (**Figure 3B**) respectively. In the medium interval, the identified modules have a recall precision of 0.79 and a precision of 0.90 with modules of 22 nodes (**Figure 3C**). The module *a priori* defined with 55 nodes has precision of 1 and recall 0.74 in the medium interval respectively (**Figure 3D**).

## References

65. Ideker T, Ozier O, Schwikowski B, Siegel AF. Discovering regulatory and signalling circuits in molecular interaction networks. Bioinformatics. 2002;18 Suppl 1: S233–S240. doi:10.1093/bioinformatics/18.suppl_1.S233

66. Maraziotis IA, Dimitrakopoulou K, Bezerianos A. Growing functional modules from a seed protein via integration of protein interaction and gene expression data. BMC Bioinformatics. 2007;8: 408. doi:10.1186/1471-2105-8-408

67. Sameith K, Antczak P, Marston E, Turan N, Maier D, Stankovic T, et al. Functional modules integrating essential cellular functions are predictive of the response of leukaemia cells to DNA damage. Bioinformatics. 2008;24: 2602–2607. doi:10.1093/bioinformatics/btn489

68. Scott MS, Perkins T, Bunnell S, Pepin F, Thomas DY, Hallett M. Identifying regulatory subnetworks for a set of genes. Mol Cell Proteomics. 2005;4: 683–692. doi:10.1074/mcp.M400110-MCP200

69. Klein PN, Ravi R. A Nearly Best-Possible Approximation Algorithm for Node-Weighted Steiner Trees. J Algorithms. 1995;19: 104–115. doi:10.1006/jagm.1995.1029

70. Ljubi?? I, Weiskircher R, Pferschy U, Klau GW, Mutzel P, Fischetti M. An algorithmic framework for the exact solution of the prize-collecting steiner tree problem. Mathematical Programming. 2006. pp. 427–449. doi:10.1007/s10107-005-0660-x

71. Lehne B, Schlitt T. Protein-protein interaction databases: keeping up with growing interactomes. Hum Genomics. 2009;3: 291–297. doi:E54180217L822J23 [pii]

72. Maier D, Kalus W, Wolff M, Kalko SG, Roca J, Marin de Mas I, et al. Knowledge management for systems biology a general and visually driven framework applied to translational medicine. BMC Syst Biol. 2011;5: 38. doi:10.1186/1752-0509-5-38

73. Mishra GR, Suresh M, Kumaran K, Kannabiran N, Suresh S, Bala P, et al. Human protein reference database--2006 update. Nucleic Acids Res. 2006;34: D411–D414. doi:10.1093/nar/gkj141

74. Margolin AA, Nemenman I, Basso K, Wiggins C, Stolovitzky G, Favera RD, et al. ARACNE: An Algorithm for the Reconstruction of Gene Regulatory Networks in a Mammalian Cellular Context. BMC Bioinformatics. 2006;7: S7. doi:10.1186/1471-2105-7-S1-S7

## Text S1 - Figure Legends

### Figure 1 - Transformation into a Steiner Arborescence problem for identifying an optimal Price Collecting Steiner Tree.

**A-C.** An example of a graph G with three nodes of weight 3, 10 and 0 and with edges of weight 2 and 7. The optimal sub-network in this graph has a score of 11 and contains the nodes scored by 3 and 10 (calculated using equation 3). In order to identify the optimal sub-network, the graph is first transformed into a Steiner arborescence problem. For this, first an artificial root R is inserted in the graph **B**. Then a direct edge from R to all other nodes in G with score higher than zero is introduced and the edge is scored using equations in **C**. Arcs between all other nodes are introduced and the cost is recomputed using equations in **C**.

**D.** A feasible arborescence with minimum total edge score corresponds to an optimal PCST, i.e. best solution.

**Figure 2 - Generation of a priori defined modules in the simulated search space.**

### Figure 3 - Accuracy of the identified modules.

**A** and **B** represent the modules pre-defined in the high MI interval respectively with 22 nodes or 55 nodes.

**C** and **D** represent the modules pre-defined in the intermediate MI interval respectively with 22 nodes or 55 nodes.


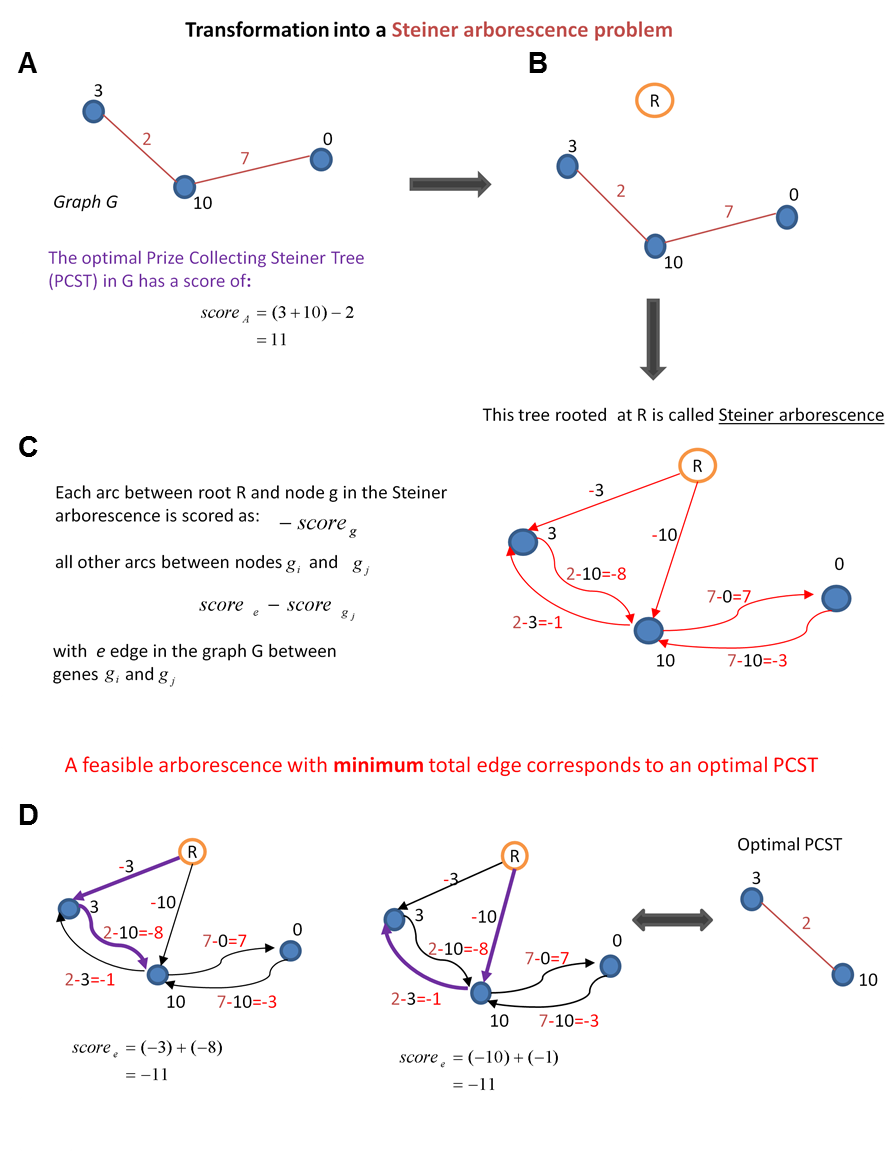


**Figure 1 - Transformation into a Steiner Arborescence problem for identifying an optimal Price Collecting Steiner Tree.**


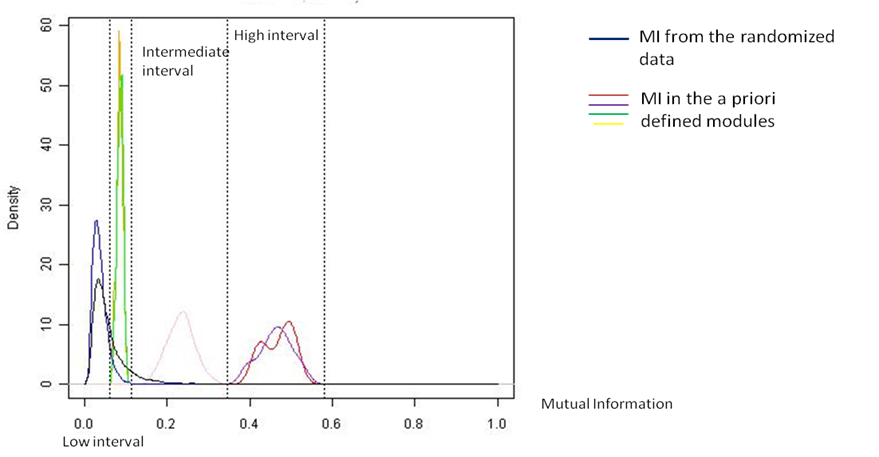


**Figure 2 - Generation of *a priori* defined modules in the simulated search space.**


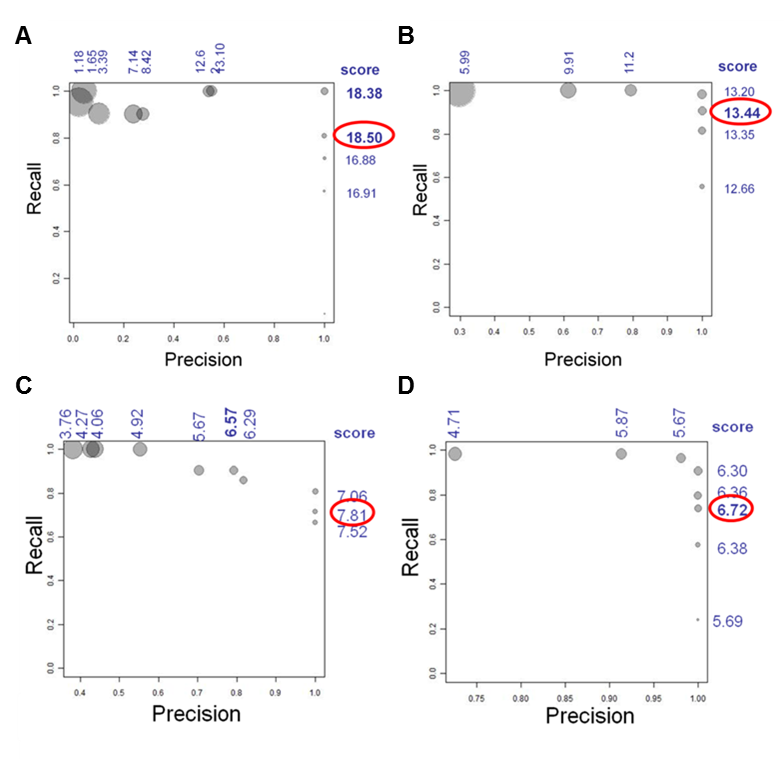


**Figure 3 - Accuracy of the identified modules.**
